# A large scale analysis of mHealth app user reviews

**DOI:** 10.1007/s10664-022-10222-6

**Published:** 2022-10-12

**Authors:** Omar Haggag, John Grundy, Mohamed Abdelrazek, Sherif Haggag

**Affiliations:** 1 Faculty of Information Technology, Monash University, Melbourne, Australia; 2 School of Information Technology, Deakin University, Melbourne, Australia; 3 School of Computer Science, The University of Adelaide, Adelaide, Australia

**Keywords:** mHealth apps, User reviews, App store, Google play, Classification, Analysis, Recommendations

## Abstract

The global mHealth app market is rapidly expanding, especially since the COVID-19 pandemic. However, many of these mHealth apps have serious issues, as reported in their user reviews. Better understanding their key user concerns would help app developers improve their apps’ quality and uptake. While app reviews have been used to study user feedback in many prior studies, many are limited in scope, size and/or analysis. In this paper, we introduce a very large-scale study and analysis of mHealth app reviews. We extracted and translated over 5 million user reviews for 278 mHealth apps. These reviews were then classified into 14 different aspects/categories of issues reported. Several mHealth app subcategories were examined to reveal differences in significant areas of user concerns, and to investigate the impact of different aspects of mhealth apps on their ratings. Based on our findings, women’s health apps had the highest satisfaction ratings. Fitness activity tracking apps received the lowest and most unfavourable ratings from users. Over half of users who reported troubles leading them to uninstall mHealth apps gave a 1-star rating. Half of users gave the account and logging aspect only one star due to faults and issues encountered while registering or logging in. Over a third of users who expressed privacy concerns gave the app a 1-star rating. However, only 6% of users gave apps a one-star rating due to UI/UX concerns. 20% of users reported issues with handling of user requests and internationalisation concerns. We validated our findings by manually analysing a sample of 1,000 user reviews from each investigated aspect/category. We developed a list of recommendations for mHealth apps developers based on our user review analysis.

## Introduction

Mobile applications have become an essential aspect of our daily lives. mHealth apps are one of the most rapidly evolving categories of mobile apps, with millions of people downloading and using some form of mHealth mobile app frequently from all over the world. According to the World Health Organisation (WHO), more than 30% of adults are deemed insufficiently physically fit due to a lack of activity and having an unhealthy lifestyle (Higgins 2016). Inactivity kills over 3.2 million people each year, according to Higgins (2016) and Joseph et al. (2014). An unhealthy lifestyle is characterised by insufficient exercise, excessive stress, poor diet, and inconsistent sleeping habits (Organisation WH 2020; Joseph et al. 2014; Spittaels et al. 2007). Solving these issues has become an essential goal for healthcare organisations and providers.

Interest in mHealth apps has grown dramatically in recent years, especially in the aftermath of the COVID-19 global pandemic and country lock-downs (Timmers et al. 2020; Pal et al. 2020; Dwivedi et al. 2020; Ventola 2014; Saadat et al. 2020). As a recent example, a whole new category of mobile apps known as“Contact Tracing” has emerged in the mobile app market.

Since a great many people use smartphones worldwide, mHealth apps have the chance to change this behaviour by motivating and encouraging people to exercise and stay healthy in their daily lives. According to WHO, 61% of the people who downloaded and started using mHealth apps have managed successfully to increase their physical activity. Moreover, the usage of mHealth apps in self-monitoring provides excellent control for people with chronic health conditions such as the elderly, which decreases the burden on health resources (Williams et al. 1998; Baker et al. 2002; Baker et al. 1998; Williams et al. 1995).

**Fig. 1:**
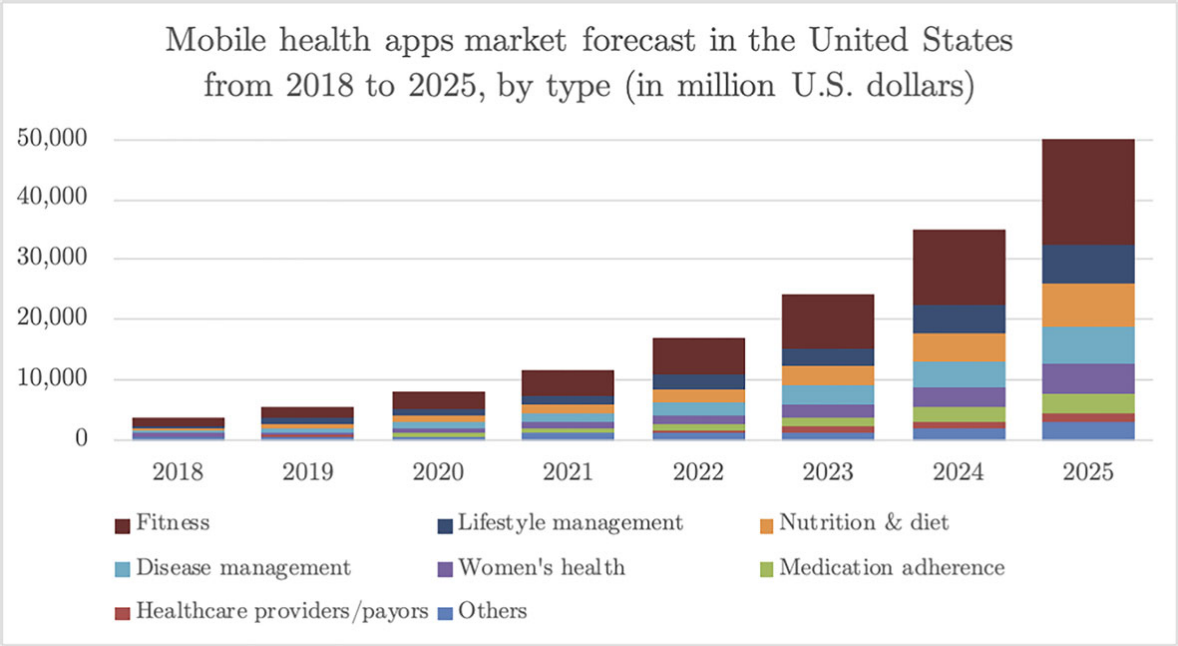
mHealth apps revenues by subcategory in US millions

The mHealth app global market is growing very fast, which includes high revenues and downloads count, as shown in Fig. 1,^1^. According to WHO, mHealth apps should be accessible to everyone, regardless of their gender, culture, age, and language. mHealth app developers should allow their apps to be accessible to the elderly, disabled, and those who are consistently identified as high-risk groups (Thelwall and Levitt 2020). Elderly and disabled people face numerous challenges and have unique needs, and it is unfair to make it difficult for them to use mHealth apps (Morey et al. 2017). Since mHealth apps save lives and improve the lives of elderly and disabled people, mHealth apps need to be designed and developed in a particular way to be fully accessible by all types of users. According to the authors of (Morey et al. 2017), it is worthwhile to invest in developing mHealth for elderly users because older adults are likely to be the largest community using mHealth apps as these apps can bring significant benefits for them. While interacting with mobile apps, users from diverse cultural backgrounds have different preferences (Oliveira et al. 2016; Reinecke and Bernstein 2011; Reinecke and Gajos 2014). Feedback from users of the same app differs across different cultures and languages (Guzman et al. 2018). Many mHealth apps are not highly rated, which identifies significant problems with these apps, and this explains why users have lots of issues and trouble while using these apps.

Despite the existence of over 318,000 mHealth apps in the mobile app market, there is little evidence of them working as they are meant to/intended for a variety of reasons (Byambasuren et al. 2018; Liew et al. 2019; Institute 2020; Nordrum et al 2016). Many mHealth apps are buggy, as demonstrated by (Athilingam and Jenkins 2018; Anderson et al. 2016), causing users to lose interest and abandon the app after a short period of time. By looking at the user reviews of mHealth apps, millions of users are raising and reporting significant issues and problems that they have experienced while using these apps. For a variety of reasons, app developers typically disregard and ignore these user reviews (Vasa et al. 2012; Packard 2015). Many user reviews are written in different languages, which make it a complex and highly challenging task for the developers of mHealth apps to manually translate, classify, and analyse all of these reviews and, indeed, to respond to those reviews by fixing any highlighted issues. Mobile app user reviews and star ratings contain much information that is useful, not only for app developers but also for other app users (Vasa et al. 2012). This information helps mHealth app developers and designers to understand and analyse user behaviour, expectations, and feedback. Furthermore, it enables them to regularly update their apps to improve the user experience (Li et al. 2018). In addition, it allows them to gain accurate insights into their users, which enables them to refine their marketing strategies and increase publicity (Vasa et al. 2012; Qiao et al. 2018).

In this paper, we analyse and categorise mHealth app user reviews and discuss the aspects of compliments and criticism mobile users have towards these apps. We present the relationship between these aspects and the apps’ rating. In addition, we manually analyse and present the most common issues and problems raised by the users for each analysed aspect. We then give some recommendations for app developers based on our deep analysis.

The key contributions of this research include:

- We carried out automated extraction and translation of over 5 million user reviews for mHealth apps;
- We carried out automated classification of these over 5 million mHealth app user reviews into 14 aspects;
- We compared users’ satisfaction levels across different subcategories of mHealth apps, identifying key areas of user problems and satisfaction;
- We validated our results via manual analysis of a sample of 1000 reviews for each classified aspect (14,000 reviews in total);
- We identified where significant issues and problems are raised in user reviews among different aspects of mHealth apps; and
- We provide a set of recommendations for mHealth app developers based on the analysed aspects of these apps in our study.

The rest of this paper is structured as follows. We first provide a motivation for our research and then detail our study approach and methodology. This is followed by a presentation of the key findings and then a discussion of implications for research and practice. We then summarise key related work to our study. We finally summarise key conclusions and directions for future research.

## Motivation

There are several ways mHealth app users can submit their feedback. In our research, we chose to work on user reviews since they have several advantages (Maalej et al. 2016). Moreover, they are considered the primary source of any available user feedback, directly linked to app features. Many problems and issues are reported and documented through mHealth apps’ user reviews. Those reported issues contain various issues in the UI (User Interface), sign-up, bugs, accessibility, privacy, and UX. Despite the huge revenue of mHealth apps, many of these issues seem to be replicated and not fixed by the released updates (Jusoh 2017; Adhikari et al. 2014; Chatzipavlou et al. 2016). As a result, some mHealth apps have low ratings (Heffernan et al. 2016). Mobile users generally have the right to access and use any mHealth apps they wish to under safe conditions. They should not expose themselves or their personal data to a risk that could lead to severe harm or consequences later on. Furthermore, mobile app engineering organisations are obliged by law and ethical agreements to ensure that their apps are accessible by all types of people (Organisation WH 2020). Since mHealth apps are essential for saving and improving the lives of people, we could potentially save billions of dollars per year by resolving those issues/problems and improving the engineering design and development practises of mHealth apps (Organisation WH 2020; Joseph et al. 2014; Spittaels et al. 2007; Higgins 2016).

In Chevalier and Mayzlin (2006); Mcilroy et al. (2017), the authors stated that user reviews could influence users’ decisions on downloading or purchasing a specific mobile app. Users tend to gain more confidence in downloading or purchasing an app if it has a high number of user reviews. If the majority of these reviews are positive, the mobile users’ trust increases. This explains why an app with few reviews or negative ratings may not succeed right away, as the majority of users will be hesitant to download or purchase this app (Khalid et al. 2014; Mcilroy et al. 2017). Moreover, user reviews can increase or decrease people’s interest and inspiration to download or engage with these apps based on previous ratings. On the other hand, from the mobile app developers’ side, user reviews allow them to have the power to enhance the reputation and credibility of their mobile apps by releasing updates that fulfil the needs of users based on their feedback and requests. Mobile app businesses and organisations that do not have many good reviews are definitely missing an excellent opportunity to grow their business and increase app downloads. While app store reviews provide potentially very beneficial information about how and what people expect from mHealth apps, comprehensive analysis of a large number of reviews that increase every day remains a major time-consuming challenge (Huebner et al. 2018).

Some research has already been done in this area by analysing the user reviews for other types of mobile apps (Guzman and Maalej 2014; Iacob and Harrison 2013; Chen et al. 2014; Gomez et al. 2015; Gu and Kim S 2015; Maalej and Nabil 2015). To the best of our knowledge, no work has yet been done to analyse mHealth apps’ user reviews on a large scale. Our research aims to provide insights to developers in identifying key issues raised by the users of mHealth apps through their user review feedback and submitted ratings. This will help them to proactively identify previously reported users’ issues before releasing new apps or updates and prevent these issues from happening in the future. Furthermore, it will assist mHealth app developers and designers in conducting better mHealth app engineering practices. By developing an automated tool that can extract, translate, and classify millions of mHealth app users’ reviews into different aspects.

By classifying the users’ reviews into different aspects and analysing them, we will be able to understand the significant issues reported by the users of mHealth apps. Moreover, we will be able to set some recommendations for app developers based on each analysed aspect. In this work, we investigate user reviews relating to fourteen mHealth app aspects – advertising, payment and subscription, compatibility, UI/UX (User Interface/ User Experience), resources, stability, connectivity, account and logging, privacy and security, user requests, notifications and alerts, uninstallation, updates, and internationalisation issues on the ratings of mHealth apps. This analysis will provide mHealth app developers comprehensive insights into the expectations and perceptions of their users when using these applications. The mHealth apps used in our study are categorised into twelve different subcategories. These mHealth app subcategories are diet and weight loss, emerging, education, women’s health, fitness activity tracking, fitness and workouts, lifestyle planner and goal tracker, scheduling and reminder apps, sleep and meditation, and mental health. This categorisation gives us the opportunity to analyse our results at an additional level of depth.

We investigated the following two research questions:

- **RQ1 - Based on user reviews, what aspects of compliments and criticisms do mobile users have for mHealth apps?** – We wanted to find out to what extent do different app aspects and app subcategories affect an app’s ratings. Users can express positive or negative assessments of different mHealth apps by submitting reviews. In this RQ, we analyse user reviews for different mHealth apps and classify them into different aspects. This allows us to identify aspects of compliments and criticisms in these reviews related to different app aspects. Additionally, we investigate user assessments and perceptions of these reviews in relation to different mHealth apps’ subcategories and how they affect star ratings.
- **RQ2 - What are the key issues and problems raised by the users for each analysed aspect?** – Different aspects have a different impact on users’ usage and ratings of mHealth apps. In this RQ, we analyse app reviews and classify them into the different aspect(s) of the mHealth app that the user is commenting on. We investigate the frequency of these issues/problems that affected their usage for different aspects and mHealth app sub-categories. This allows us to identify frequent issues and problems raised by the users and give some evidence-based recommendations to handle these issues. Our findings will allow developers to ensure better practises for mHealth apps design and development.

## Study Design

### mHealth Apps Dataset and Subcategories

**Fig. 2:**
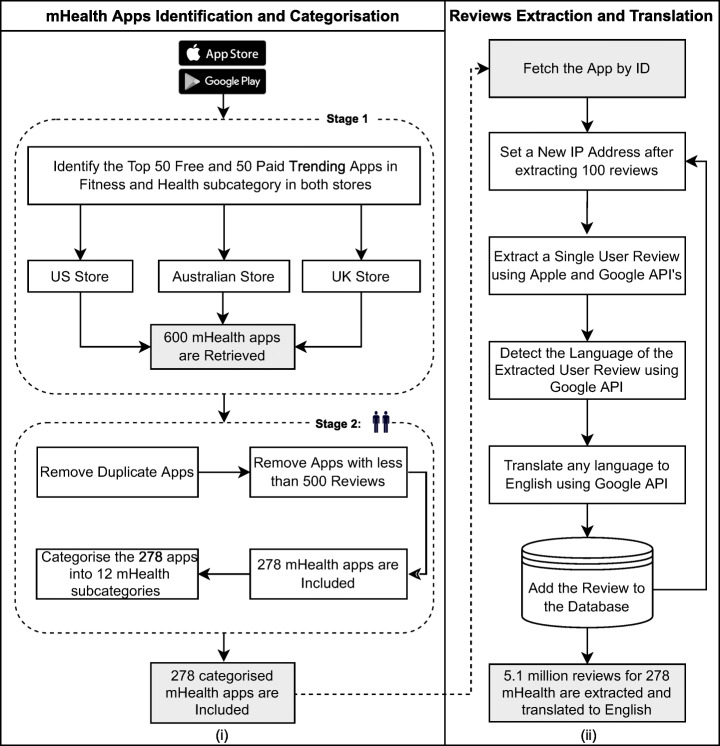
(i) mHealth Apps Identification and Categorisation Methodology (ii) Reviews Extraction and Translation Methodology

Our mHealth apps identification and categorisation process consisted of two stages, as shown in Fig. 2(i). In the first stage, to ensure diversity and inclusion, we extracted a list of the top trending 50 free apps under the fitness and health subcategory from both the Apple and Google Play stores. These trending lists are made by Apple and Google based on several factors such as apps download rate and usage. This was implemented on both app stores in three countries which are Australia, the United States and the United Kingdom. Three hundred apps were identified as a result of this process. We repeated the same process on paid apps resulting in a total of 600 apps.

In the second stage, we removed the common apps between the Apple and Google Play stores to avoid repetitive apps in our analysis. Finally, we removed any app that had fewer than 500 user reviews as the goal of our study to identify the major issues occurring in the most commonly used mHealth apps. The whole procedure resulted in having user reviews for 278 mHealth apps, in which we have analysed the reviews of these apps. We wanted to see if different mHealth apps have fundamentally similar or different user reviews for different app characteristics. To discuss our work at an additional level of depth, we manually re-categorised all mHealth apps retrieved in our research into sub-categories such as fitness activity tracking and women’s health apps, as shown in Table 1. The subcategories of mHealth apps used in our study were based on this study (Bach and Wenz 2020).

**Table 1:** mHealth app subcategories used in our user reviews classification ratings

mHealth apps subcategory	Apps that...
Fitness activity tracking	... allow users to track their physical activities or movements,
	e.g.: “Strava: Track Running, Cycling & Swimming” and “Fitbit”.
Fitness and workouts	... allow users to access fitness workouts and exercises, e.g.:
	“Home Workout - No Equipment” and “Mindbody: Home
	Workout & Fitness App”
Diet and weight loss	... allow users to access weight or improve their eating regimen,
	e.g.: “Simple: Fasting & Meal Tracker” and “Lose It!
	– Calorie Counter”
Education and Information	... allow users to access medical information and advice, e.g.:
	“Reflectly: Self-care journal” and “Motivation - Daily quotes”
Mental Health	... allow users to adjust or improve their mental health, e.g.:
	“Moodistory Mood Tracker, Diary” and “Replika: My AI Friend”
Sleep and meditation	... allow users to improve their sleeping or relaxation habits, e.g.:
	“Moshi: Sleep and Mindfulness” and “Sleep Booster - Sleep Better”
Patient health tracking	... allow doctors to track, e.g.: “Instant Heart Rate:
and self-monitoring	HR Monitor” and “HeartWatch: Monitor Heart Rate”
Women’s health	... allow women to improve their general health and habits, e.g.:
	“Flo: Health & Period Tracker” and “Clue Period & Cycle Tracker”
Emerging	... are released by developers in a short time span as a result
	of unexpected event such as COVID-19 apps, e.g.:
	“COVIDSafe” and “NHS COVID-19”
TeleHealth and telemedicine	... allow users to have online appointments with their doctors
	and access their prescription, e.g.: “HealthEngine” and “Well
	Pharmacy NHS prescription delivery”
Lifestyle Planner and	... allow users to change their lifestyles and set goals, e.g.: “I’m
Goal Tracker	Done Drinking” and “Shibboleth Lifestyle Journal”
Scheduling and reminders	... allow users to schedule or make reminders to healthy activities, e.g.:
	“WaterMinder” and “Workout Calendar - Motivation”

Moreover, we wanted to be able to understand how these aspects differ across mHealth app sub-categories. This allows us to explore the role of different aspects such as privacy, user interfaces, and sign-up experiences of mHealth apps to provide comprehensive insights into users’ perceptions of their mHealth apps

### Investigated App Aspects in User Reviews

In our previous work (Haggag et al. 2021), we manually analysed over 23,000 user reviews in both Apple and Google Play stores belonging to different apps’ categories such as Health, Social Media, Emerging, etc. Our goal was to create a list of keywords that can be used to identify different aspects of an app that users are commenting on. The analysed mHealth app aspects were adapted and modified from a previous study done on finance apps (Huebner et al. 2018). We determined that there are many problems and issues reported and submitted by users of mHealth apps, as shown in Table 2.

**Table 2:** App aspects used in our user reviews classification (adapted from Haggag et al. 2021 and Huebner et al. 2018)

App Aspect	A user review containing...
Privacy	... privacy or security related issues, e.g collecting or accessing
	users data, information, location, etc.
Stability	... stability or failure related issues, e.g. crashes, freezes, bugs, etc.
Advertising	... ads or commercials related issues, e.g frequent ad banners,
	pop-ups, etc.
Requests	... user requests related issues, e.g. feature, bug fix, app updates
	requests, etc.
Uninstallation	... users deleting or uninstalling the app because of privacy, stability issues, etc.
Payments	... billing or subscriptions related issues, e.g in-app purchases, premium upgrades, etc
Compatibility	... OS or HW related issues, e.g. unsupported versions, OS requirements,
	unsupported external devices, etc.
Resources	... resources related issues, e.g. battery drainage, memory usage, etc.
Connectivity	... connectivity or networks related issues, e.g. Bluetooth, 3G,
	4G, WiFi, NFC, etc.
Account and	... accounts or logging related issues,
Logging	e.g. sign up, login, logout , etc.
Resources	... resources related issues, e.g. battery drainage, memory usage, etc.
Notification	... notifications or alerts related issues, e.g. too frequent or few
	alerts, notifications, etc.
Updates	... app updates related issues, e.g. bugs or software issues after
	updates, etc.
Internationalisation	... internationalisation related issues, e.g. language, culture,
	countries, etc.

These problems are grouped into errors in UI (User Interface), sign-up, bugs, accessibility, privacy, and UX (User Experience) related issues. Identifying these aspects helps us to specify how various aspects of mHealth apps influence their ratings. Moreover, we wanted to be able to understand how these aspects differ across mHealth app sub-categories. This allows us to explore the role of different aspects such as privacy, user interfaces, and sign-up experiences of mHealth apps to provide comprehensive insights into users’ perceptions of their mHealth apps.

### Automated App Reviews Analysis

We developed an analysis tool written in Python that automatically extracts, translates and classifies mHealth app user reviews. Our tool uses a bag of keywords classification approach to classify and investigate how different aspects of mHealth apps can affect their ratings. Below we discuss in detail how our automated tool works.

- The tool first uses GooglePlay and AppleStore open APIs to extract user reviews. If a review is not written in English, it uses the Google Translate API library (Translate 2021) to detect the language of all the reviews and translate them into English.
- The tool then preprocesses each review by (i) correcting misspelt words, (ii) performing stopwords, and (iii) stemming the text.
- It then classifies each review as addressing zero or more of our 14 different app aspects. To do this we use the keywords chosen based on our work manually analysing over 23,000 user reviews (Haggag et al. 2021).
- Finally, the tool automatically generates various statistics for each app subcategory, app aspect, appstore, and overall.

**Step 1 – Review Extraction and Translation**: First, we pass to our tool a list containing the IDs of the apps for which we want to extract the reviews from both GooglePlay and AppleStore. The tool then uses GooglePlay and AppleStore open APIs to extract user reviews from both platforms. For each extracted review, we get the following information: review date, App ID, App Name, Country, App version, Author (user name), rating (from 1 to 5 stars), title of the review, and finally, the review itself.
  We use Google Translate API (Translate 2021) to detect the language and translate the extracted review if it was not already in English. We had some challenges since we were detecting the language and translating millions of reviews from different languages.
  To the best of our knowledge, Google Translate is the most accurate free translation service that allows translations from any language. Google uses its own neural machine translation technology as its translation approach. It uses deep learning techniques to translate entire sentences at a time without having to provide any input for the language. Google Translate’s accuracy varies by language, but it has an overall accuracy level of more than 82.7% (Translate 2021). For higher efficiency, if the detected language of the review is in English, it is added directly to the database. Otherwise, the review is written in another language, so our tool translates it into English. Then the tool adds the translated review to the database. Figure 2(ii) illustrates our tool methodology to extract and translate user reviews.
**Step 2 – Review Categorisation**: Our tool now extracts each review from the database and classifies each one as the user commenting on zero or more of our 14 different app aspects. We use the keywords developed based on our previous manual analysis of over 23,000 user reviews from different types of apps (Haggag et al. 2021). Our tool uses a bag of keywords classification approach to determine whether a review is relevant to each specific aspect or not. To begin, the tool preprocesses each review by (i) correcting misspelt words, (ii) performing stopwords, (iii) generating synonymous, and (iv) stemming the text. The tool employs an autocorrect spell checker library API (fsondej 2021) to correct misspelt words. The tool uses the NLTK library’s vocabulary and algorithms (NLTK 2021) to apply stopword removal and stemming.
  A step we took to improve the performance of the classification process was to remove all non-related tokens/words from the reviews before classification. To improve our classification accuracy, we used an online Thesaurus API (thesauruscom 2021) to generate all synonyms for the word’s first definition if the words in a review did not match any of the keywords used in our aspects classification. This helped us to classify more reviews according to our defined set of 14 app aspects. Finally, if the analysed reviews contain one of the keywords of interest, the tool classifies the review into the relevant aspect. We did not employ machine learning-based algorithms to classify relevant reviews since there was no labelled dataset available online for the analysis of mHealth apps, according to our knowledge.
  For example, the following review will be classified to the “advertisements” aspect since it has the word ads:

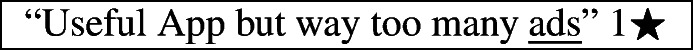
  The following review will be classified to the “privacy” aspect since it has the word privacy:

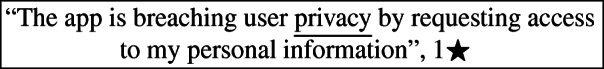
  The following review will be classified to the “privacy” aspect since it has the word scam:

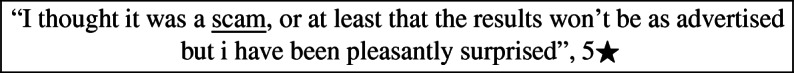
  The following review will be classified to the “Internationalisation” aspect since it has the word English.

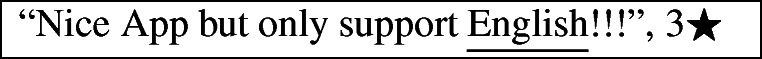
**Step 3 – Statistical Analysis**: Our tool applies statistical analysis to generate a set of statistics for the given input file. This includes the count of the total number of reviews per aspect per app. Moreover, it counts the number of reviews both for each aspect and for each star rating. In addition, it combines and adds the statistics of two or more apps together, which results in having the stats for each mHealth app subcategory.

**Fig. 3:**
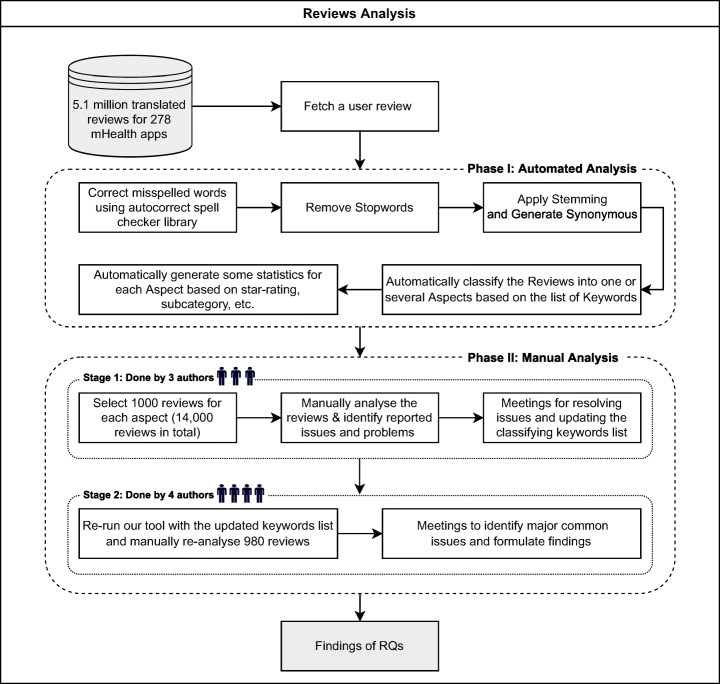
Our manual and automated user reviews analysis methodology

Figure 3 illustrates the tool processing. The number of analysed user reviews and the overall rating for each mHealth are shown in Table 3.

**Table 3:** Overall number of analysed user reviews and apps for each mHealth app subcategory

mHealth App Subcategory	Number of Reviews	Number of Apps
Fitness Activity Tracking	1,379,126	64
Fitness and Workouts	1,420,762	47
Diet and weight Loss	644,584	39
Education and Information	216,429	23
Mental Health	113,818	11
Sleep and Meditation	558,997	34
Patient Health Tracking & Self-monitoring	159,798	21
Women’s Health	1,083,157	31
Emerging	39,082	5
TeleHealth and Telemedicine	19,387	12
Lifestyle Planner and Goal Tracker	155,038	17
Scheduling and Reminders	80,673	11

### Step 4 – Manual App Review Analysis

After the automated extraction, translation and classification of over 5.1 million user reviews for 278 mHealth apps into 14 aspects and to identify the significant issues that were found in each analysed aspect, we manually analysed 1,000 reviews randomly selected for each aspect. This manual review analysis step consisted of two stages, as shown in Fig. 3.

In the first stage, to ensure diversity and inclusion, we applied stratified random sampling to those 1,000 selected reviews from each aspect. The class was based on the star rating, (200) 1-star reviews, (200) 2-stars reviews, (200) 3-stars reviews, (200) 4-stars reviews, and (200) 5-stars reviews, which is statistically significant at 95% confidence level (CL) and 5% margin of error (ME). This procedure resulted in the manual analysis of 14,000 reviews in total. Our manual review analysis is based on reading the whole review word-by-word and then checking if the review fits into one of our 14 current aspects. If the review keywords did not fit any of the analysed aspect word lists or were wrongly classified, we carefully considered one of the following two options. Option 1, adding a new keyword to our list so reviews with this term will be classified into the right category. Alternatively, option 2, which is removing the word that led to the wrong classification. The decision on each was taken in several physical and virtual meetings between the authors to achieve consensus.

In stage 2, we ran our tool again with the updated keywords list and manually re-analysed 980 reviews that had conflicts from stage 1. These enhancements improved the overall accuracy of our classification from 84% to 91%.

## Findings

Using our automated analysis tool, we have automatically extracted, translated, and classified over 5.1 million user reviews sentences for different subcategories of mHealth mobile apps into 14 different aspects. These reviews were extracted from both Apple and Google Play stores belonging to 278 mHealth apps. Our tool uses a ”bag of keywords” classification approach to classify and investigate how different aspects of mHealth apps can affect their ratings. In addition, all mHealth apps used in our study were manually sub-categorised into 12 different subcategories. We used the star rating of the app and the app review classification to identify strengths and weaknesses in the apps. From our manual analysis of 23,000 app reviews, we identified that most app reviews mention only 1 or 2 app aspects, allowing us to correlate app rating and app aspect(s) mentioned. Then we did a manual analysis of 14,000 user reviews (1,000 reviews for each aspect) to identify the major issues that were found in each analysed aspect and to provide evidence-based recommendations for the developers of mHealth apps.

**Fig. 4:**
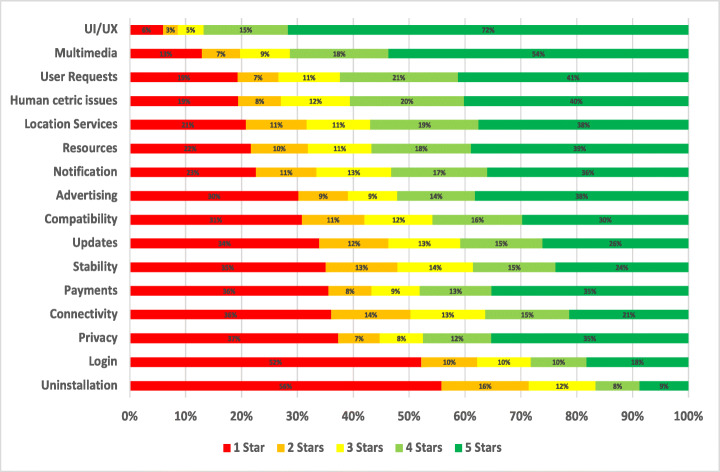
Distribution of star ratings across all aspects (percentages shown in bars)

The distribution of the star ratings of each analysed aspect in our study is depicted in Figure 4 and analysed further below. The top raised aspects in all mHealth apps included in our study were UI and UX (28.81%), User Requests (13.44%), Payments/Subscription (8.54%), Stability (7.55%), Compatibility (5.77%), Updates (5.44%), and Connectivity (3.48%). The uninstallation aspect reviews were the worst rated, followed by login and then privacy. On the other hand, the UI/UX aspect was the best-rated, followed by multimedia and then user requests.

The uninstallation aspect was the worst-rated, with 55.82% of the users who raised uninstallation issues in their review rated the app with only one star. Followed by account and logging issues where 52.2% of the users rated the app with only one star. Then, privacy issues where 37.36% of the users who raised privacy issues rated the app with only one star. On the other hand, UI/UX was the best-rated aspect, where only 6% of the users rated it 1 star, followed by internationalisation issues, where 19.46% of the users rated it 1 star, and then user requests, where 19.33% of the users rated the app 1 star.

As the most highly rated subcategory, women’s health app users were the most satisfied. In contrast, fitness activity tracking was the lowest-rated app subcategory in our assessment of all the mHealth subcategories. Due to several common issues and problems, users gave fitness activity tracking applications low ratings on both the App Store and Google Play.

In the following subsections, we identify the most positive and negative aspects across different sub-categories of mHealth apps based on classified user reviews. We also determine what are the key issues and problems raised by the users for each analysed aspect.

### UI and UX

**Fig. 5:**
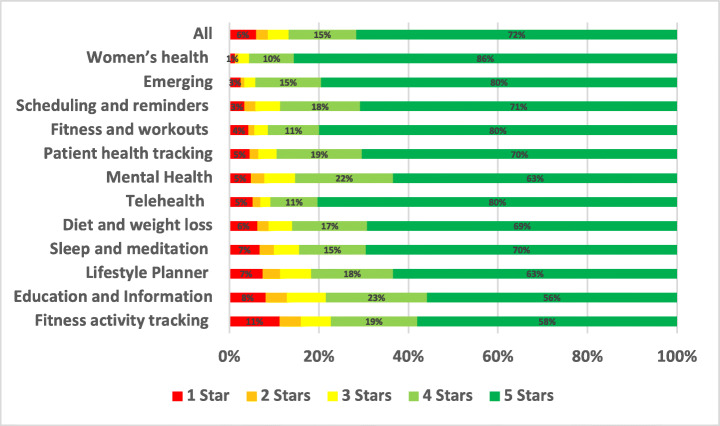
Overall percentage for UI and UX aspect across subcategories

User Interface and User Experience issues were the most common issues, reported in 28.81% of the total user reviews analysed in our study. A summary of the overall percentages for UI and UX aspect across different mHealth subcategories is shown in Fig. 5. Note that a large majority of the app reviews mentioning UX features are linked with positive (four and five star) rated apps. This indicates that unlike some other app aspects, raising UX issues is generally a positive mention.

TeleHealth and telemedicine apps were the most highly affected by UI and UX issues with 54.67% of all their reviews mentioning UX issues, followed by Emerging apps with 52.55% and then Education and Information apps with 33.11%. On the other hand, the least mHealth sub-categories mentioning UI and UX issues in their reviews were Mental Health apps at 21.7%, followed by Sleep and meditation apps with 23.37% and then Fitness activity tracking apps with 26.86%. By looking into the overall percentage for UI and UX aspect across sub-categories of mHealth apps, Fitness activity tracking apps were the worst-rated, where 11.22% of the users who raised UI and UX issues in their reviews rated these apps with only 1 star. Followed by Education and Information apps, where 8.17% of the users rated these apps with only 1 star. Some mentioned UX issues are illustrated below.

#### App Design

Having a great UX design for mHealth apps directly affects users’ satisfaction and leads users to enjoy the app and share their positive experiences with others by writing a positive review. By analysing user reviews, the developers of mHealth apps are successfully addressing users’ concerns and avoiding the most common mistakes regarding UX issues. This behaviour led to a high star rating from mHealth apps’ users for this aspect compared to other aspects included in our study. The users also reported some concerns regarding poor navigation and unclear content in the design of some apps. These reported issues include insufficient colour contrast, inappropriate text or button sizes, unresponsive buttons, or unexpandable menus. Moreover, it is reported that the UI in some apps is overcrowded, which affects users’ usage of these apps and makes the apps more complex to navigate. Another issue that was reported is that some mHealth apps use the same interface as mobile webpages. However, the app does not work properly compared to the website. For example: 
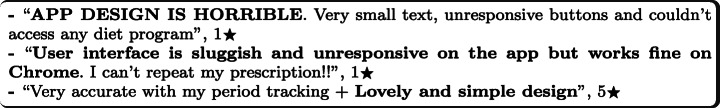


#### Usability and Users satisfaction

Usability of the app and users’ satisfaction are directly correlated in our manually analysed user reviews. The more the mHealth app was usable, the more users were satisfied and submitted high ratings. The mHealth apps’ design, content, and functionality enable users to achieve their goals and express their satisfaction levels. User reviews reveal that when mHealth app developers provide users with more information or functionalities than they actually need, some users will become frustrated and may not use these apps. Women’s mHealth apps were the best rated for UX as women see them as useful, straightforward and have a simple UI and navigation. Users raised in their user reviews that some mHealth apps have long loading times or are slow while being used, affecting their experience using the app. Others reported low satisfaction levels as the apps had poor navigation or content. In general, most mHealth apps’ subcategories have high satisfaction levels and good UX. For example: 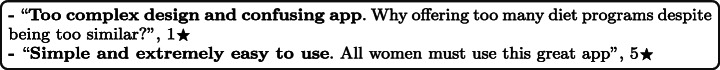


### Users Requests

**Fig. 6:**
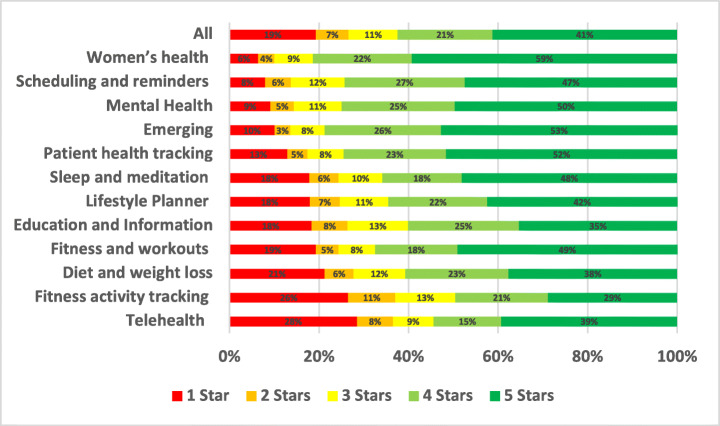
Overall percentage for user requests aspect across subcategories

Users Requests were reported in 13.44% of the total user reviews analysed in our study. A summary of the overall percentage for users requests aspect across different mHealth subcategories is shown in Fig. 6.

Education and Information apps were the most highly affected by user requests issues with 22.98% of all of their app reviews including them, followed by Emerging apps with 21.92% and then Lifestyle Planner and Goal Tracker apps with 21.05%. On the other hand, the least mHealth sub-categories affected by users requests issues were Women’s health apps with 7.49% of their user reviews mentioning them, followed by Fitness and workouts apps with 10.23% and then Patient health tracking and self-monitoring apps with 13.16%. By looking into the overall percentage for users requests aspect across subcategories of mHealth apps, teleHealth and telemedicine apps were the worst-rated where 28.49% of the users who raised users requests issues in their reviews rated these apps with only 1 star. Followed by fitness tracking apps, where 26.47% of the users rated these apps with only 1 star. Examples of such reviews are illustrated below.

#### Feature and enhancement requests

Hundreds of thousands of mHealth apps exist nowadays in the mobile app markets. To keep competitive, app developers need to listen to the requests received by their users. By analysing users’ reviews, we found out that users constantly request additional features to be implemented within the apps or ask for some enhancements. Surprisingly, some of these requests were repetitive and not addressed in later updates, which clearly shows that either the developers of these apps are not reviewing the users’ reviews or they do not have the will to address them for some reason. Failing to address these requests leads users to get frustrated and switch to another app or even delete the app. For example: 
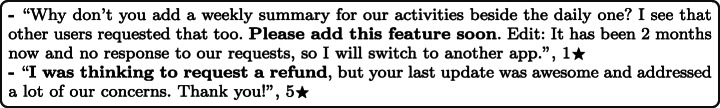


#### Bug fix and update requests

Before an update to mHealth apps is released to the public, most of them pass a quality assurance (QA) phase. Unfortunately, having a QA phase does not mean that all users will accept the app or ensure that it is free from any bugs or issues. By manually analysing user reviews, we found out that some bugs still exist after developing updates, although users were requesting this bug to be fixed in earlier versions. This leads users to keep asking for enhancements and bug fixes in later versions. Moreover, users keep asking app developers to develop and add some features and functionalities during their updates. Since every user has their own needs, user requests to add features and functionalities differ from one user to another. For example: 
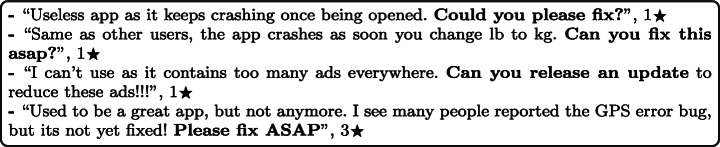


### Payments

**Fig. 7:**
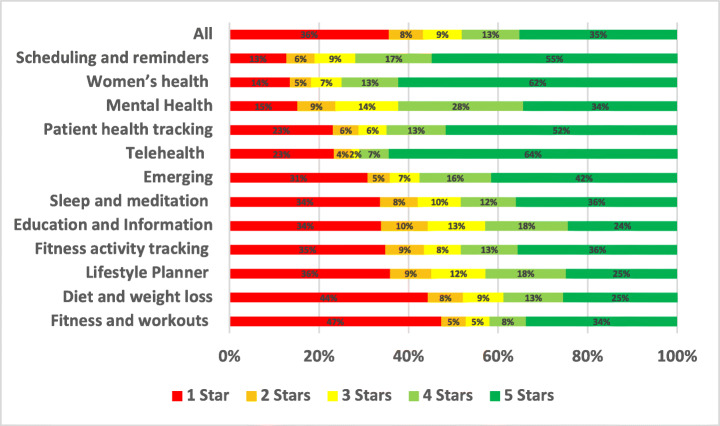
Overall percentage for payments aspect across subcategories

Payment and subscription issues were reported in 8.54% of the total user reviews analysed in our study. A summary of the overall percentage for Payments aspect across different mHealth subcategories is shown in Fig. 7.

Education and Information apps were the app categories whose user reviews mention Payments issues, with 23.72% of all reviews, followed by Lifestyle Planner and Goal Tracker apps with 21.08% and then Sleep and meditation apps with 17.68%. On the other hand, the least mHealth sub-categories with reviews mentioning payment and subscription issues were Emerging apps with 0.58%, followed by TeleHealth and telemedicine apps with 2.35% and then women’s health apps with 2.44%. By looking into the overall percentage for payments aspect across subcategories of mHealth apps, Fitness and workouts apps were the worst-rated where 47.31% of the users who raised Payments issues in their reviews rated these apps with only 1 star. Followed by Diet and weight loss apps, where 44.29% of the users rated these apps with only 1 star. Some of the payment issues mentioned in app reviews are illustrated below.

#### Apps functionality and In-app purchases

Simply because mobile users can download some mHealth apps for free does not mean they will not have to pay for them later. In-app purchases can quickly pile up and encourage users to spend money on add-ons, subscriptions, premium features, and other items. In-app purchases are available in all categories of apps on both the App Store and Google Play. It was reported in the reviews that some users were unsatisfied when they installed an mHealth app for free and it did not allow them to use the important features and functionalities written in the description since they were using the free version. In addition, they can not remove the apps’ ads unless they pay for the premium version of the app. Moreover, some users reported that they were charged for these ostensibly optional expenses on their credit cards without their knowledge. For example: 
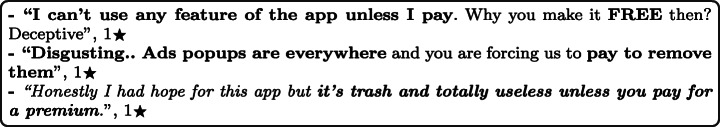


#### Billing and refund

In-app purchase is considered any amount of money an app may request beyond the initial cost of downloading the app. Many in-app purchases are voluntarily made to provide new features; others are subscriptions that require users to sign up and pay a price in order to be able to use the app. Having a credit or debit card linked with the App Store or Google Play account is required to cover any expenses related to installing paid apps or requesting an in-app purchase feature. Since mobile stores allow apps to charge the card linked with the account directly, in-app purchases are dangerously simple to make, as there’s no need to enter credit card information or, in many cases, even a password. These ostensibly optional expenses can be charged to credit cards without the cardholder’s knowledge. It was reported in the reviews that some of the users were charged for using an in-app purchase feature. However, they did not request this feature or initiate any payments. Moreover, others who had already purchased in-app features found out that the app either did not unlock the premium features even after paying or, in some cases, they were charged for the same feature multiple times. For example: 
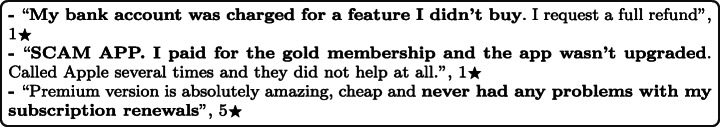


### Stability

**Fig. 8:**
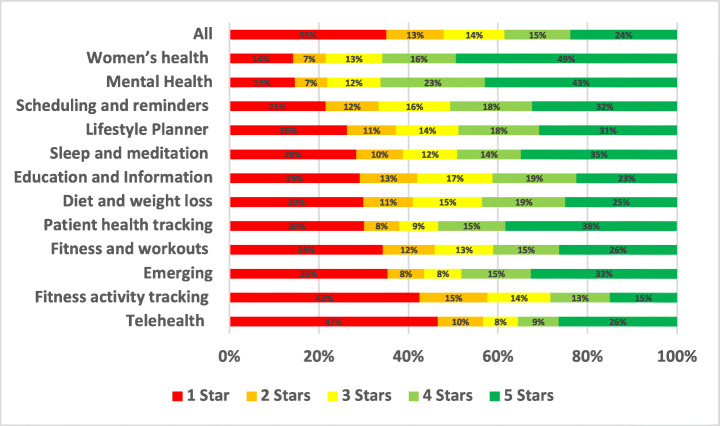
Overall percentage for stability aspect across subcategories

Stability issues were reported in 7.55% of the total user reviews analysed in our study. A summary of the overall percentage for stability aspect across different mHealth subcategories is shown in Fig. 8.

Fitness activity tracking apps were the most highly affected by stability issues with 14.42% of all of their reviews mentioning them, followed by emerging apps with 10.02% and then Education and Information apps with 9.39%. On the other hand, the least mHealth sub-categories affected by stability issues were women’s health apps with 3.12% of their reviews mentioning these issues, followed by fitness and workouts apps with 4.05% and then diet and weight loss apps with 5.48%. By looking into the overall percentage for stability aspect across subcategories of mHealth apps, TeleHealth and telemedicine apps were the worst-rated where 46.57% of the users who raised stability issues in their reviews rated these apps with only 1 star. Followed by fitness tracking apps, where 42.49% of the users rated these apps with only 1 star. Some example stability issues mentioned in mHealth app reviews are discussed below.

#### Bugs

Most of the stability issues highly reported across different subcategories of mHealth apps were caused by bugs. The types of bugs reported by users of mHealth apps in their user reviews range in severity. Some of them were minor, and others were more severe. It was reported in the user reviews that it affected the apps’ performance and even led the whole app to crash after performing a specific action in the app, such as tapping a specific button, which disturbs their usage and affects the usability of these apps. While others reported some less severe stability issues where the portrait and landscape orientation of some of these apps is incorrect and not the same across different devices, since the page layouts are different across different screen resolutions and phone models. It was also reported some minor bugs, such as permission issues, notification push and logging in or signing up issues. For example: 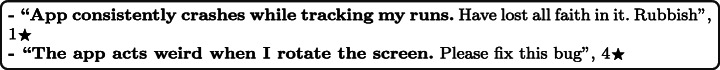


#### Developer updates and OS firmware

Many of the users of mHealth apps reported in their user reviews that there was a direct link between the released updates and stability issues, with some users reporting stability issues after updating to the latest version. Some app updates affect the stability of their phones after updating to the latest version, particularly when app developers release significant updates to add a new feature or change the app’s interface. For example: 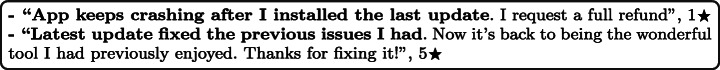


### Compatibility

**Fig. 9:**
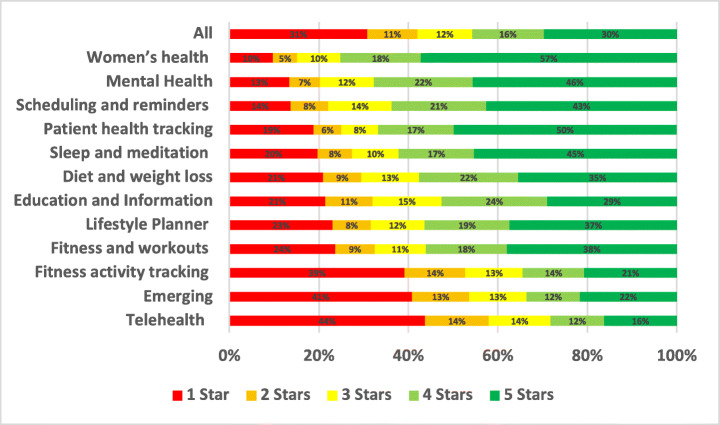
Overall percentage for compatibility aspect across subcategories

Compatibility issues relate to challenges users have in running the app on their mobile handset. Compatibility issues were reported in 5.77% of the total user reviews analysed in our study. A summary of the overall percentage of the compatibility aspect across different mHealth subcategories is shown in Fig. 9.

Fitness activity tracking apps were the most highly affected by compatibility issues, mentioned in 12.47% of all of their user reviews, followed by patient health tracking and self-monitoring apps with 7.98%, and then Education and Information apps with 6.33%. On the other hand, the least mHealth sub-categories affected by compatibility issues were women’s health apps with 1.38% of their user reviews mentioning such issues, followed by Mental Health apps with 2.32% and then fitness and workouts apps with 2.42%. By looking into the overall percentage for compatibility aspect across subcategories of mHealth apps, TeleHealth and telemedicine apps were the worst-rated where 48.07% of the users who raised compatibility issues in their reviews rated these apps with only 1 star. This was followed by newly emerging mHealth apps, such as COVID-19 related apps, where 41.18% of the users rated these apps with only 1 star. Some examples of such issues are given below.

#### External devices

Many of the app user reviews reported that there exist some problems with connecting and linking external devices or trackers, such as smartwatches to mHealth apps. These problems were usually caused since these external devices are not fully supported, outdated, or interfere with other external devices. Some comments are highly negative, some highly positive, depending on the app’s (lack of) support for these external devices. For example: 
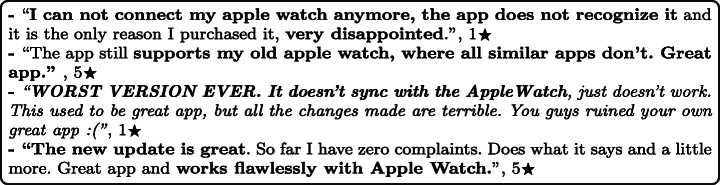


#### Mobile firmware versions

The firmware version of the operating system plays a key role in app compatibility issues. It was reported by many users that some mHealth apps are not compatible with old versions of mobile firmware. The users reported that some of the developers’ updates affect the compatibility of their apps with their devices, so they need to update their phones to the latest firmware to be able to use these apps. Users who own old phones that do not support the latest OS firmware have also reported that they can no longer use the app after these updates, and these apps have become entirely inaccessible to them. For example: 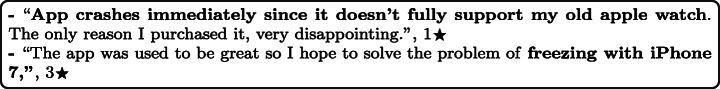


### Updates

**Fig. 10:**
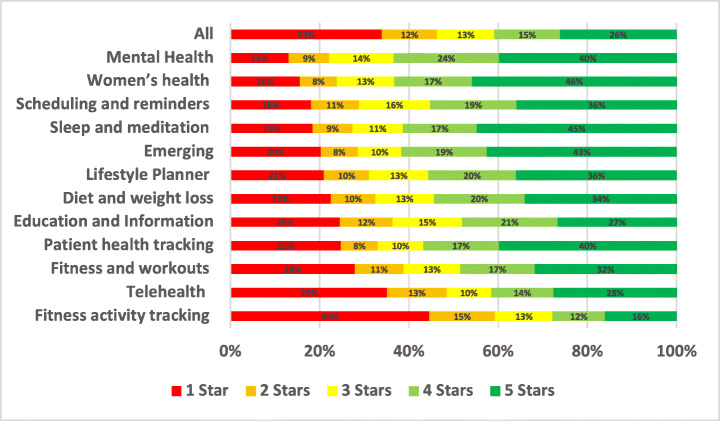
Overall percentage for updates aspect across subcategories

App update issues were reported in 5.44% of the total user reviews analysed in our study. A summary of the overall percentage for updates aspect across different mHealth subcategories is shown in Fig. 10.

Fitness activity tracking apps were the most highly affected by updates issues with 10.92% of all reviews mentioning them, followed by Education and Information apps with 7.60% and then Patient health tracking and self-monitoring apps with 5.12%. On the other hand, the least mHealth sub-categories affected by updates issues were women’s health apps with 1.92% user app reviews mentioning them, followed by Fitness and workouts apps with 2.21% and then Mental Health apps with 3.45%. By looking into the overall percentage for updates aspect across subcategories of mHealth apps, Fitness activity tracking apps were the worst-rated where 44.58% of the users who raised updates issues in their reviews rated these apps with only 1 star. Followed by TeleHealth and telemedicine apps apps, where 35.14% of the users rated these apps with only 1 star. Examples of such issues are illustrated below.

#### Issues occurring after updates

Most mHealth apps pass through a testing phase before being released to the public. When developers develop some updates to add some features or enhancements to the app or fix some bugs, they can actually create more trouble than benefits for some users through the updated app. During our manual analysis of the users’ reviews for mHealth apps, it was highly reported that users who updated their apps are either highly satisfied or not. That is why 33.93% of the users rated the apps with only 1 star and only 26.15% rated the apps as five stars. This reveals that quality assurance engineers are not highly verifying the mHealth apps’ quality before releasing them to the users. For example: 
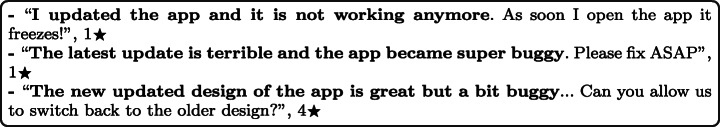


#### Users requesting updates

mHealth apps offer some features and functionalities to allow users to improve their health. Every user has their own needs, and depending on the functionality of the mHealth app; they decide if they want to download and continuously use it or not. By manually analysing users’ reviews, it was reported that users are requesting app developers to develop some updates to add some features and functionalities, fix some bugs or increase accessibility. Surprisingly, some of these user requests were repetitive among some users, and the app developers did not address them in the released updates. For example: 
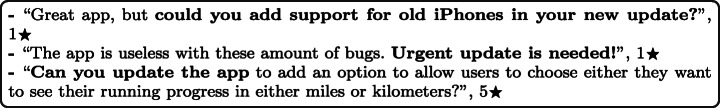


### Connectivity

**Fig. 11:**
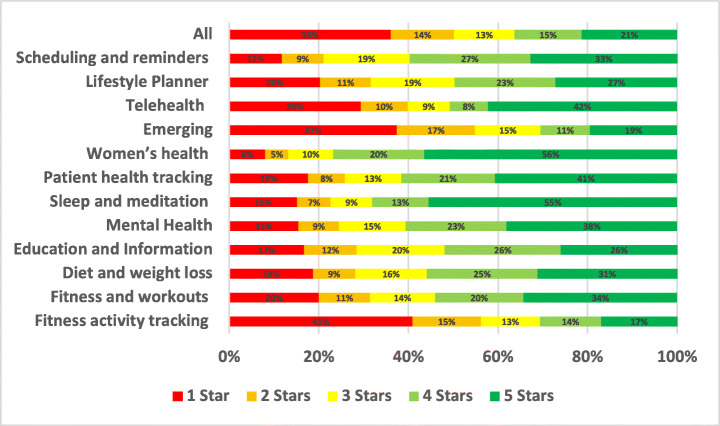
Overall percentage for connectivity aspect across subcategories

Connectivity issues were reported in 3.48% of the total user reviews analysed in our study. A summary of the overall percentage for connectivity aspect across different mHealth subcategories is shown in Fig. 11.

Education and Fitness activity tracking apps were the most highly affected by connectivity issues with 11.28% of all of their user app reviews reporting such issues, followed by Emerging apps with 3.32% and then Patient health tracking and self-monitoring apps with 1.66%. On the other hand, the least mHealth sub-categories affected by UI and UX issues were Women’s health apps with 0.27% of their user reviews mentioning such issues, followed by Lifestyle Planner and Goal Tracker apps with 0.59% and then Scheduling and reminders apps with 0.85%. By looking into the overall percentage for connectivity aspect across subcategories of mHealth apps, Fitness activity tracking apps were the worst-rated where 40.93% of the users who raised connectivity issues in their reviews rated these apps with only 1 star. Followed by emerging apps, where 37.42% of the users rated these apps with only 1 star. Examples of these issues are presented below.

#### Internet Connection

Many mHealth apps need either to connect to the internet or to communicate with other devices when in use. Apps may encounter issues that cause their users’ connections to be lost, disconnected, or run at slow speeds. Users may not detect or even comprehend the existence of connectivity problems when using the app, which results in compounding the problem. By analysing users’ reviews, it was reported that some apps are dependent on the internet to function, and when the network connection is broken, they become useless. Others reported that they do not get any notifications about the time when the app will have maintenance, so they are aware that they can not use the app at all during this period. Furthermore, some users reported that the same app behaved differently on different devices. For example, a user has reported that the connection of his mHealth app with the internet was not successful, although the same app works fine on his other phone, and he uses the same network on both devices. For example: 
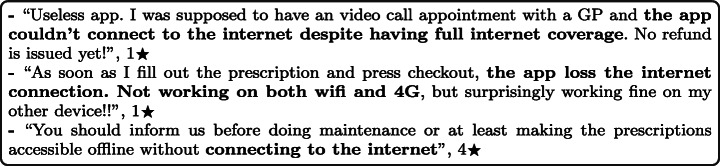


#### Device Connectivity

Some mHealth apps, especially within the fitness activity tracking subcategory, allow users to connect and link external devices using Bluetooth. This enables users to track and monitor their physical activity accurately, enhancing usability and improving the user’s experience. By analysing users’ reviews, it was reported that some mHealth apps do not fully support external devices such as smartwatches, which causes issues with users not being able to use these devices. Moreover, it was reported that developer updates could lead to connectivity issues, where some users reported that their external devices became unsupported after updating their apps. Users say that they should be aware of all the supported external devices before downloading or purchasing the apps. In addition, app developers should consider testing different external devices before releasing new app updates. For example: 
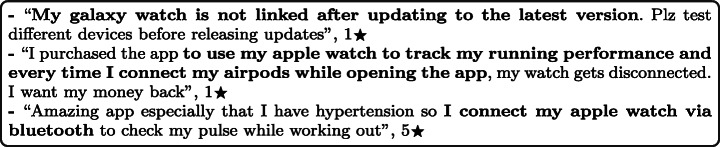


### Internationalisation

**Fig. 12:**
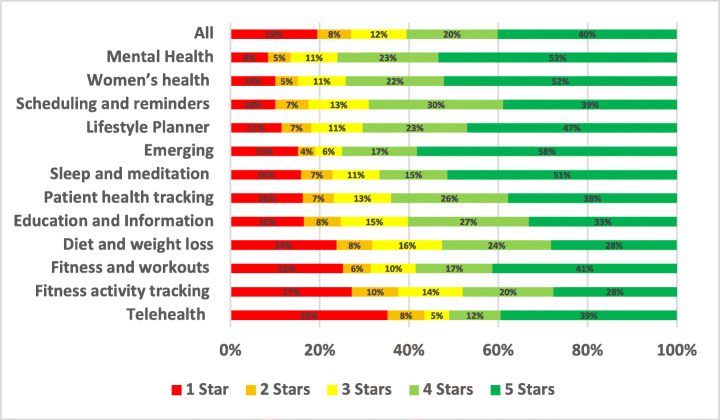
Overall percentage for internationalisation aspect across subcategories

Internationalisation issues were reported in 2.25% of the total user reviews analysed in our study. A summary of the overall percentage for updates aspect across different mHealth subcategories is shown in Fig. 12.

Mental Health apps were the most highly affected by internationalisation issues with 8.16% of all reviews mentioning them, followed by Education and Information apps with 4.60% and then Lifestyle Planner and Goal Tracker apps with 4.26%. On the other hand, the least mHealth sub-categories affected by internationalisation issues were women’s health apps with 1.10% user app reviews mentioning them, followed by Fitness and workouts apps with 1.34% and then TeleHealth and telemedicine apps with 1.79%. By looking into the overall percentage for internationalisation aspect across subcategories of mHealth apps, TeleHealth and telemedicine apps were the worst-rated where 35.16% of the users who raised internationalisation issues in their reviews rated these apps with only 1 star. Followed by fitness tracking apps, where 27.2% of the users rated these apps with only 1 star. Examples of such issues are illustrated below.

#### Language

For users of mHealth apps, not being able to utilise the app in their native language is a significant problem. When users can use the app in their own language, they feel more comfortable, according to user reviews. mHealth app users reportedly demanded that apps be made available in their native tongue, according to reviews. It is also worth noting that users grumbled when the app did not support their native language. Others claimed that the interface and features of the app differed depending on the language. In addition, several users have noticed that the translation has a few inaccuracies. Other users reported that they are glad that the app supports the language they speak. 
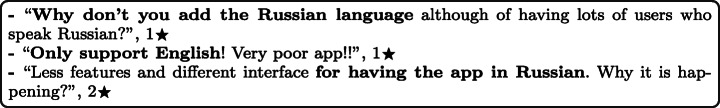


### Account and Logging

**Fig. 13:**
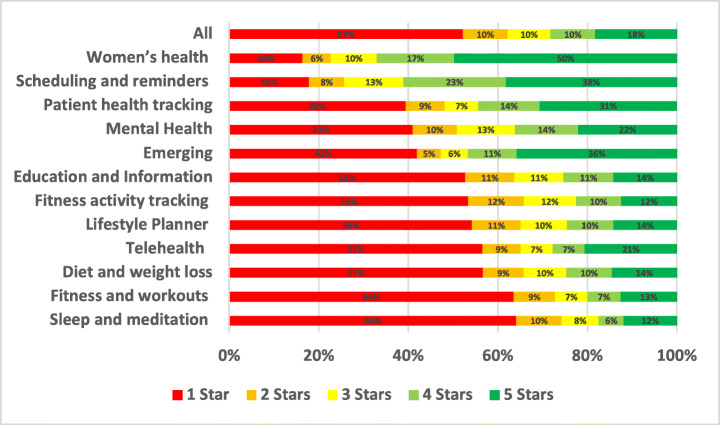
Overall percentage for login aspect across subcategories

Login issues were reported in 2.23% of the total user reviews analysed in our study. A summary of the overall percentage for login aspect across different mHealth subcategories is shown in Fig. 13.

Emerging apps were the most highly affected by account and logging issues with 12.47% of all of their user reviews mentioning such issues, followed by TeleHealth and telemedicine apps with 7.36% and then Education and Information apps with 4.44%. On the other hand, the least mHealth sub-categories affected by login issues were Women’s health apps with only 1.01% of their app user reviews mentioning these issues, followed by Mental Health apps with 1.17% and then Scheduling and reminders apps with 1.58%. By looking into the overall percentage for login aspect across subcategories of mHealth apps, sleep and meditation apps were the worst-rated where 64.13% of the users who raised login issues in their reviews rated these apps with only 1 star. Followed by Fitness and workouts apps, where 63.51% of the users rated these apps with only 1 star. Examples of such issues are illustrated below.

#### Sign up

Many mHealth apps require users to sign up and create an account to use the app. This allows developers to give users a customised experience while using these apps and will enable them to have better security. By analysing user reviews, it was reported that the signup process could be challenging for some users since it is poorly executed, which makes these apps inaccessible. We have compiled a list of the most common issues raised by mHealth app users in their reviews:

- Complex signup forms where users need to complete and submit too many details or information.
- Buggy signup forms where users face errors while entering their data within the registration form.
- Verification codes are not delivered to the email where users can not access the confirmation link to confirm their account and complete the registration process.
- OTP (One-time password) codes are not delivered to mobile numbers where users can not complete the registration process.
- Incorrect verification codes where users get errors when they submit the verification code they have received.
- Since some phone number formats are not supported, some users may encounter errors when entering their phone numbers. This issue caused these users not to be able to complete the signup process to use the app.

A few representative examples of such issues in reviews include: 
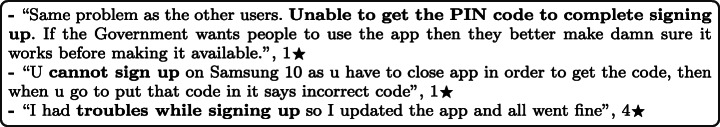


#### Sign in

Most mHealth apps require users to log in to their accounts to use the app entirely. This allows app developers to enhance the security of their apps and protect users’ data. By analysing users’ reviews, the following issues were highly reported by the users:

- Users forgot their users’ names, and they could not retrieve them. This issue leads users to not log in to the app anymore and force them to create a new account.
- Forgot passwords functionality is not working properly where users can not reset their password if they forgot it. This issue is caused by either getting an error while filling out the reset password form or not receiving the reset email.
- Apps send an OTP (One-time password) to the mobile number of the users where users do not have access to this number either temporarily or permanently.
- The app keeps signing out, requiring users to log in each time, causing a great deal of inconvenience.
- Users are trying to log in while the app is temporarily down. This issue happens when users receive no notification about the problems and outages of the app, so they reset their passwords; however, this does not solve the issues, and they are still not able to log in.

A few representative examples of such issues in reviews include: 
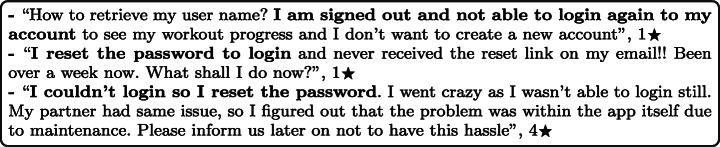


### Notification

**Fig. 14:**
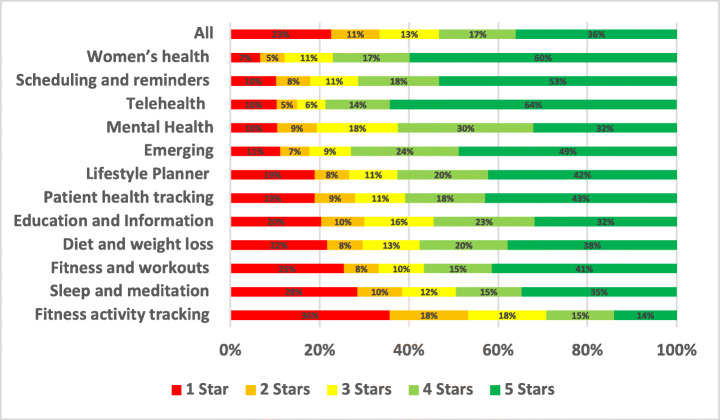
Overall percentage for notification aspect across subcategories

Notification issues were reported in 2.15% of the total user reviews analysed in our study. A summary of the overall percentage for notification aspect across different mHealth subcategories is shown in Fig. 14.

Scheduling and reminders apps were the most highly affected by notification issues with 13.71% of all of their app reviews mentioning them, followed by Emerging apps with 4.99% and then Lifestyle Planner and Goal Tracker apps with 4.64%. On the other hand, the least mHealth sub-categories affected by notification issues were Fitness and workouts apps with 1.07% of ther user reviews mentioning them, followed by Mental Health apps with 1.22% and then Sleep and meditation apps with 1.49%. By looking into the overall percentage for notification aspect across subcategories of mHealth apps, Fitness activity tracking apps were the worst-rated where 35.7% of the users who raised notification issues in their reviews rated these apps with only 1 star. Followed by Sleep and meditation apps, where 28.44% of the users rated these apps with only 1 star. Examples of such issues are illustrated below.

#### Notification Frequency

App notifications are often used to convey reminders, updates, and other critical information about the app’s performance and demand special attention or further action from users. According to our user review analysis, notifications are considered a double-edged sword for app developers and users. Push notifications can help developers increase users’ satisfaction if they are useful, timely, personalised and relevant. Otherwise, they may appear to be annoying, useless and can lead users to delete the app. Some apps send out too many notifications to users, including premium app subscription offers, which mHealth app users find annoying and distracting. Other users have written in their reviews some compliments about how the app’s notifications, reminders and alerts were helpful. For example: 
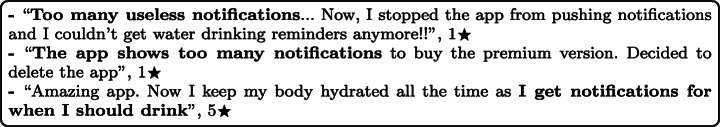


#### Notification Content

mHealth apps’ users are usually satisfied when they receive notifications that are highly tailored to them. According to user feedback, tailored and helpful notifications are generally well-received by users. It was clear that some mHealth apps’ developers managed to gather enough information about their users’ preferences through their use of their app to adapt the messaging to different users. Several complaints were lodged against app developers who sent the same notification to the same users in the app. Our user review analysis revealed the importance of how app developers need to make sure that the content of their app’s notifications will be unique and dependent on the user’s interaction with the app. For general announcements and to avoid annoying users with irrelevant notifications, app developers can ask users to opt-in for a weekly or monthly generic non-personalised notification. For example: 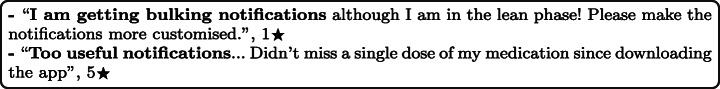


### Privacy

**Fig. 15:**
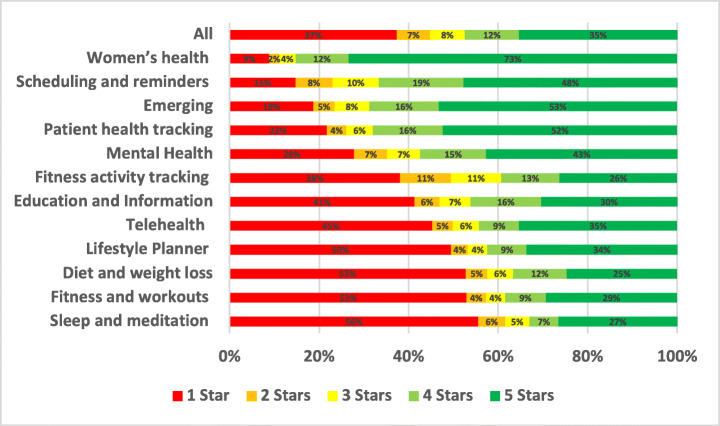
Overall percentage for privacy aspect across subcategories

Privacy issues were reported in only 1.23% of the total user reviews analysed in our study, a somewhat surprising result given the surge in interest in app data privacy in recent years. A summary of the overall percentage for privacy aspect across different mHealth subcategories is shown in Figure is shown in Fig. 15.

Emerging apps were the most highly affected by privacy issues with 4.43% of all of their app reviews mentioning these issues, followed by Education and Information apps with 2.11% and then Fitness activity tracking apps with 1.93%. On the other hand, the least mHealth sub-categories affected by privacy issues were Fitness and workouts with 0.74% of their app reviews mentioning such issues, followed by Women’s health apps with 0.76% and then Sleep and meditation apps with 0.91%. By looking into the overall percentage for privacy aspect across subcategories of mHealth apps, Sleep and meditation apps were the worst-rated where 55.55% of the users who raised privacy issues in their reviews rated these apps with only 1 star. Followed by Fitness and workouts apps, where 52.92% of the users rated these apps with only 1 star. Examples of these issues are illustrated below.

#### Users’ Privacy

Many mHealth apps need their users to allow some permissions to enable them to use the apps. Some permissions request accessing users’ sensitive information such as location, gallery, contacts or interaction with other apps. Most of these permissions jeopardise users’ privacy because they are only granted once and continue to access data until the user terminates them. Moreover, some mHealth apps can log and track all users’ activities with incorrect permissions. Worryingly, according to the analysis of user reviews, many users are granting these tracking permissions within their mobile apps without understating what is happening behind the scenes. Many users are perplexed as to why their mHealth apps request these permissions, although they are not required for their core functionality. The following were the most frequently raised privacy concerns in user reviews:

- Apps pasting user’s most recently copied text once they are opened.
- Apps accessing users’ locations in the background.
- Apps gaining access to users’ contacts.
- Apps gaining access to users’ cameras.
- Apps gaining access to users’ photo galleries.
- Apps gaining access to users’ microphones.
- Apps gaining access to users’ other devices that are connected to the local network.
- Apps gaining access to users’ Bluetooth.
- Apps tracking users’ interaction with other apps.
- Apps asking for users’ private information such as date of birth.
- Apps forcing users to accept their lengthy and complicated privacy policies before they can use.

Some representative examples of such app reviews include: 
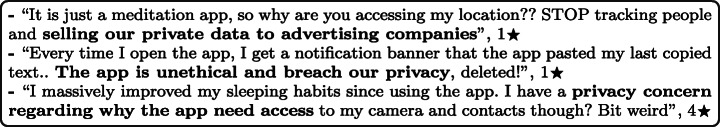


### Uninstallation

**Fig. 16:**
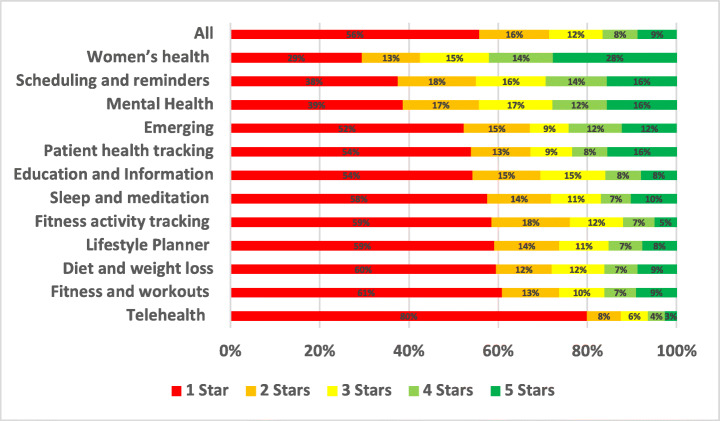
Overall percentage for uninstallation aspect across subcategories

Uninstallation issues were reported in 1.09% of the total user reviews analysed in our study. A summary of the overall percentage for uninstallation aspect across different mHealth subcategories is shown in Fig. 16.

Emerging apps were the most highly affected by uninstallation issues with 3.61% of all of their user app reviews mentioning such issues, followed by Fitness activity tracking apps with 1.97% and then TeleHealth and telemedicine apps with 1.36%. On the other hand, the least mHealth sub-categories affected by uninstallation issues were women’s health apps with 0.37% of their app reviews mentioning them, followed by Fitness and workouts apps with 0.61% and then Patient health tracking and self-monitoring apps with 0.69%. By looking into the overall percentage for uninstallation aspect across subcategories of mHealth apps, TeleHealth and telemedicine apps were the worst-rated where 79.92% of the users who raised uninstallation issues in their reviews rated these apps with only 1 star. Followed by Fitness and workouts apps, where 60.83% of the users rated these apps with only 1 star. Examples of such issues are illustrated below.

#### Apps containing severe bugs or inaccessible

Typically, developers of mHealth apps want to create apps that are both stable and feature-rich in order to increase app adoption. Failure to accomplish that goal may result in users deleting or uninstalling these apps. According to manual analysis of user reviews, one of the primary reasons for users to delete or uninstall the app is the app’s frequent bugs and stability concerns. According to reviews, several of these vulnerabilities manifest themselves only following app updates. Severe accessibility flaws that significantly impair users’ ability to use the app can also cause them to delete it. According to some users, significant accessibility flaws stopped them from using the app, prompting them to delete it. For example: 
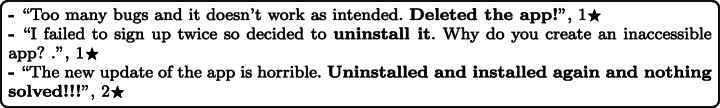


#### Apps are not as described

The description of mHealth apps should be informative to allow people to know the application’s functionality and features before downloading it. By analysing user reviews carefully, users generally delete or uninstall the app when they find out that the app is not fulfilling their expectations. One reason for users to uninstall mHealth apps has been identified as having an mHealth application that needs some in-app purchases to unlock major functionalities and features in the app. This happens usually as the app developers do not properly explain the specific features of the free edition in the app description. Furthermore, several users remark that app screenshots appearing in the app description do not match reality. For example: 
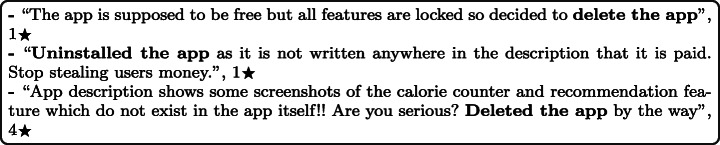


### Advertising

**Fig. 17:**
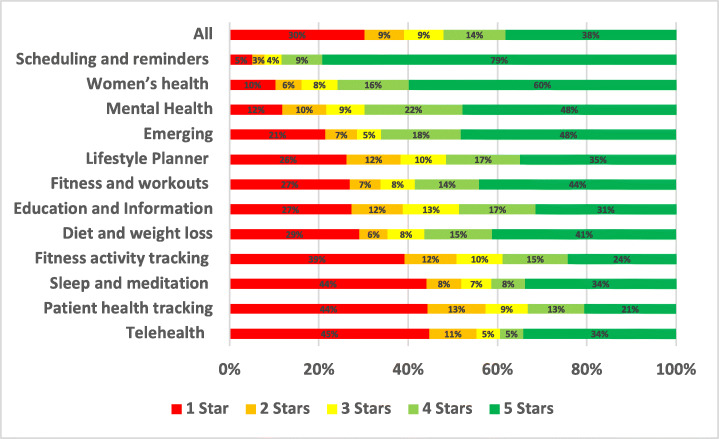
Overall percentage for advertising aspect across subcategories

Advertising issues were reported in only 0.97% of the total user reviews analysed in our study. A summary of the overall percentage for advertising aspect across different mHealth subcategories is shown in Fig. 17.

Patient health tracking and self-monitoring apps were the most highly commented on regarding advertising issues, with 2.23% of all of their app reviews mentioning them, followed by Fitness and workouts apps with 1.19% and then Lifestyle Planner and Goal Tracker apps with 1.15%. On the other hand, the least mHealth sub-categories with reviews mentioning advertising issues were emerging apps with 0.14%, followed by TeleHealth and telemedicine apps with 0.2% and then Mental Health apps with 0.48%. By looking into the overall percentage for advertising aspect across subcategories of mHealth apps, TeleHealth and telemedicine apps were the worst-rated where 44.74% of the users who raised advertising issues in their reviews rated these apps with only 1 star. Followed by Patient health tracking and self-monitoring apps apps, where 44.33% of the users rated these apps with only 1 star.

#### Ads frequency

There are several mobile app monetisation strategies. In-app advertising is the most common strategy to monetise many free mHealth apps i.e. selling advertising banner appearances in the app. In-app mobile ads are a huge money generator, and many mobile app developers depend on such ads to increase their overall profits. Despite the importance of these advertisements to the developers of these apps, it has been reported in user reviews that these ads can be very annoying, distracting and may even lead to the deletion of these apps if they are too frequent. Moreover, app developers and publishers are accused of being guilty of prioritising their own business needs over the interests of their users. When it comes to apps with a high ad frequency, users express a lot of dissatisfaction in the reviews. In addition, non-skippable or too lengthy ads were frequently complained about in the user reviews. For example: 
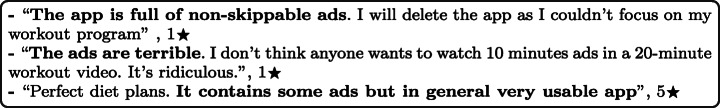


#### Ads content, style and app functionality

Mobile advertising is an excellent option for many app developers to keep their apps free for users while also generating some profits. There should be a balance between the app developers’ monetisation goals and the users’ requirements for whatever value your app provides. Even if the mHealth app’s general design is flawless, the content of the ads or how they are placed and presented can drive users away. The types of mobile ads reported in the analysed user reviews were banner, interstitial, video or rewarded video ads. Inappropriate or unrelated ads were highly reported in user reviews as some users reflected their dissatisfaction with the content of the ads shown. Others claim that ad placement or position affects their app usage, causing the app to freeze or hide some menus or buttons in the app interface. On the other hand, users have expressed their satisfaction with rewarding video ads, as they can gain some points or tokens to help them unlock some premium features of the app after watching these videos. For example: 
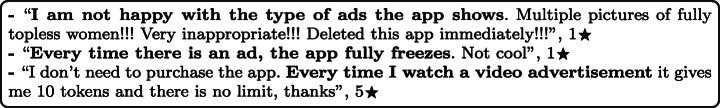


### Resources

**Fig. 18:**
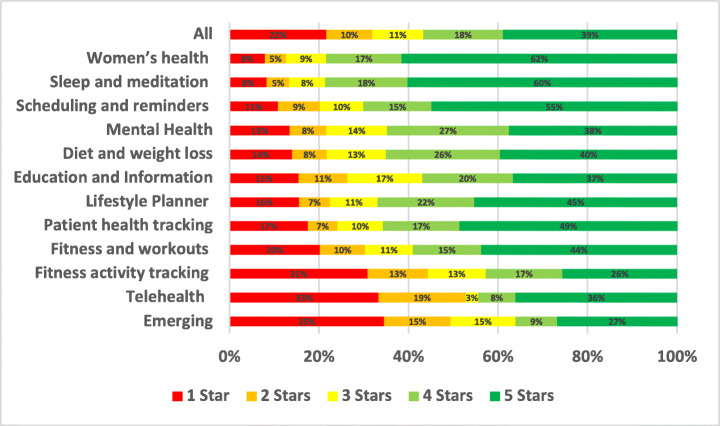
Overall percentage for resources aspect across subcategories

Resources issues were reported in 0.75% of the total user reviews analysed in our study. A summary of the overall percentage for resources aspect across different mHealth subcategories is shown in Fig. 18.

Emerging apps were the most highly affected by resources issues with 3.73% of all of their user reviews reporting such issues, followed by Sleep and meditation apps with 2.16% and then Fitness activity tracking apps with 1.67%. On the other hand, the least mHealth sub-categories affected by resources issues were TeleHealth and telemedicine apps with 0.19% user reviews mentioning such issues, followed by Fitness and workouts apps with 0.19% and then women’s health apps with 0.21%. By looking into the overall percentage for resources aspect across subcategories of mHealth apps, Emerging apps were the worst-rated where 34.59% of the users who raised resources issues in their reviews rated these apps with only 1 star. Followed by TeleHealth and telemedicine apps apps, where 33.33% of the users rated these apps with only 1 star. Examples of such issues are illustrated below.

#### Battery

Fast drainage of mobile batteries is one of the most common and frustrating issues that mobile users face while using some mHealth apps. Mobile users usually try to solve this issue by cutting the usage of some apps and accessing the apps’ websites through the browser instead. This method has worked as a workaround for users frustrated with battery drain, especially for social media apps. By analysing the users’ reviews, we found that battery drainage issues were common in apps that require long usage by the users. Some users who use fitness activity tracking apps have reported that they need to use a bulky power bank so their phone does not die during their exercise because they use a lot of processing power, such as the GPS (Global Positioning System). Others link the relationship between apps generally tracking or checking users’ location or these apps that work in the background all the time and send the users frequent notifications. Due to battery drainage issues, some mHealth app users have deleted these apps or limited what these apps can do with their phones. For example: 
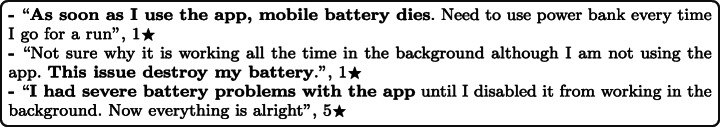


#### Memory and Storage

Every mHealth app has its own specifications and requirements. Some apps require ample free space to be installed and work properly. Others consume a high amount of memory during usage. By analysing the users’ reviews, we found that users who own old phones or have a small storage capacity were more prone to facing resource issues while using mHealth apps. It was reported in the user reviews that some apps take a large capacity of phone storage, where users reported that this issue persists and becomes even worse after app updates. Others reported that they could not do any app updates as they got an update error message while updating, since the phone did not have enough space to perform the update. This issue has become even worse for mHealth apps that allow users to download audio and video content to access this data while being offline. mHealth apps’ users who own old phones with low computing power face huge issues while using the apps, such as slow processing, lagging and freezing. For example: 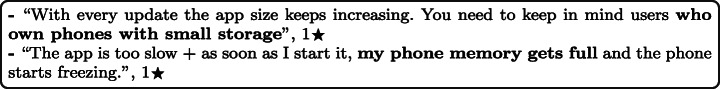


## Discussion

### Implications for Practitioners

#### UI/UX

Good mHealth app UX design is essential as mhealth app users are often under a lot of stress when using e.g. managing serious health condition, concerns with health and fitness etc. mHealth app users are also very diverse with the whole population potentially being users of an app. According to our analysis, the majority of mHealth apps analysed generally provide high satisfaction and decent UX. Key problems flagged are that some apps have poor navigation and incomprehensible text, e.g. using medical jargon or overly complex language. The inability to enlarge menus or change font sizes are other key issues reported. Several apps’ UIs are crowded, impacting user experience and navigation. Users complain when mHealth app developers present them with more information or functionality than they need. Our manual review study links app usability and user satisfaction. Some mHealth apps are slow to load or use, affecting the user experience. TeleHealth and telemedicine, Emerging, and Education and Information apps were the most highly affected subcategories by UI/UX issues from our user app review analysis. Table 4 summarises key issues with UI/UX from our automated and manual user review analysis, and some recommendations for improved UI/UX in mHealth apps based on this user feedback.

**Table 4:** Key issues and recommendations for mHealth app UI/UX issues

Key issues found	Recommendations
- Developers not implementing important	mHealth app users often use apps under very
UI requests by elderly/disabled users	stressful conditions e.g. managing severe illness
	– key UI/UX issues reported need to be priori-
	tised by developers
- Changing app interface frequently or	- Implementing simple designs for mHealth
implementing complex designs	apps
- One interface for all users	- mHealth users are very diverse – need to al-
	low users to switch between / configure inter-
	face depending on their diverse needs and ex-
	periences
- Issues with old firmware versions and	- Many mHealth users have old handsets – de-
updates	velopers need to test UI thoroughly on differ-
	ent devices with different operating systems,
	screen sizes, etc before releasing updates

#### User Requests

Our analysis indicates users regularly request new features or improvements in their mHealth apps. We found that many requests were not addressed in later versions, indicating that the developers of these apps either do not read user reviews or do not have the desire to fulfill these requests. Failing to respond to these requests frustrates users, who abandon or delete the app. As above, mHealth app users are very diverse and use the apps under stressful mental and physical conditions. Most mHealth app updates go through QA before being released to the public. Unfortunately, a QA phase does not guarantee that all users will approve the app or that it is bug-free. Education and Information, Emerging, and Lifestyle Planner and Goal Tracker apps were the most highly affected subcategories by user requests issues from our user app review analysis. Table 5 summarises key issues with user requests from our automated and manual user review analysis, and some recommendations for handling user requests in mHealth apps based on this user feedback.

**Table 5:** Key issues and recommendations for mHealth app user requests issues

Key issues found	Recommendations
- Developers ignoring and not address-	- Some mHealth apps require frequent updates
ing commonly reported issues in sub-	addressing repetitive feature and enhancement
sequent updates	requests submitted by users to remain useful
	e.g. Goal planning, Lifestyle tracking, Educa-
	tion and response by developers is essential
- mHealth apps not thoroughly tested,	- Testing the app thoroughly and ensuring the
which leads to many users’ complaints	quality assurance (QA) phase before releasing
and requests	updates to the users as these are health-critical
	apps – they are improving and saving lives
- mHealth apps not adhering to up-	- mHealth apps may become out-of-date when
dated health advice/treatments	new medical research findings are released and
	need to be proactively updated to ensure they
	are not unsafe/give wrong advice/implement
	wrong interventions

#### Payments

Although many mHealth apps are free to download, users may later have to pay for features in them or to keep them activated. While many in-app purchases are optional, some require users to subscribe and pay a fee to access the important features of the app, such as accessing detailed diet plans, joining online fitness workout classes, access more detailed plans, etc. With in-app purchases, mHealth users can easily accumulate premium features, subscriptions, etc. After downloading a free mHealth app, some mHealth users were disappointed and frustrated when they found out that they could not access the key features and functionalities offered in the app description unless they pay. In particular, in-app purchases are dangerously easy to make because they do not require credit card information and unknown charges can be made to cards, which puts them under more stress. Apparently, some mHealth app users were charged for using an in-app purchase feature they did not request by joining a free trial and then the mHealth app continue to auto-bill them. Also, some users who had paid for in-app features discovered that the app either did not unlock these features or charged them multiple times. Fitness activity tracking, Fitness and workouts, and Diet and weight loss apps were the most highly affected subcategories by payment issues from our user app review analysis. Table 6 summarises key issues with payments from our automated and manual user review analysis, and some recommendations for improved payments in mHealth apps based on this user feedback.

**Table 6:** Key issues and recommendations for mHealth app payments features

Key issues found	Recommendations
- Hidden app costs/Nontransparent to	- The description of any mHealth app should
use full functionality of mHealth apps	clearly state if it requires extra cost to use all
	functions before users become dependent on it
	for their health needs
- Incorrect and unclear mHealth app	- mHealth apps may be free for a certain trial
free vs paid description	period to allow users to test functionality be-
	fore purchasing the app and becoming depen-
	dent on it
- Sometimes viewing or analysing	- Users should be able to receive a refund using
mHealth app user records leads to	a simple form and process
unauthorised payments where users	
are wrongly charged as an in-app pur-	
chase feature	
- Charging users after trialing some	- Automatically cancelling free trials and/or
mHealth features although users are no	reminding users to cancel their free trials for
longer using it	health features before being charged
- Users are unable to unlock the pre-	- Careful testing to ensure that users who pur-
mium mhealth features even after pur-	chased the app will have no issues to unlock
chasing or subscribing to the premium	all premium mhealth functionalities
version	
- mHealth users are unable to restore	- Careful testing to have an in-app-purchase
in-app-purchasing after changing their	restore button in a clear place in the app
mobile or using a different device	

#### Stability

mHealth apps need to be well implemented and tested since they are used by diverse users, under stressful conditions, many users have accessibility and other challenges, and different users may want to use different functionalities of these apps. However, bugs cause most of the reported stability issues across mHealth app subcategories. The degree of issues reported by users of mHealth apps varies. According to user reviews, some mHealth app users were highly annoyed when stability issues slowed down the apps’ speed and caused them to crash following a specific action, such as starting a specific fitness workout, using a new feature, etc. Others experienced less serious stability issues where some apps’ portrait and landscape orientations were inaccurate and not consistent across devices due to varying page layouts for different screen resolutions and phone models, making the app hard to use for them. Mild bugs such as authorisation issues, notification push, and logging in or signing up were also noted. Some users of mHealth apps had stability concerns after updating to the latest version, according to user reviews, which led them to be unable to continue the fitness progress they were previously making using these apps. Moreover, some app updates affect phone stability, especially when app developers issue major updates to introduce new features or change the app’s design to make the app accessible to more users. Fitness activity tracking, Emerging, and Education and Information apps were the most highly affected subcategories by stability issues from our user app review analysis. Table 7 summarises key issues with stability from our automated and manual user review analysis, and some recommendations for handling stability issues in mHealth apps based on this user feedback.

**Table 7:** Key issues and recommendations for mHealth app stability issues

Key issues found	Recommendations
-users encounter bugs while analysing	- Developers need to proactively analyse user
or processing their mHealth data	reviews and addressing key reported bugs to
	ensure health dependent end users are not se-
	riously impacted
- Apps crashing/freezing when process-	- Ensuring the existence of enough computa-
ing and mining specific mHealth tasks	tional power based on the users data size; test-
	ing practices need to include range of realistic
	mHealth app user datasets and loadings
- Some mHealth functionalities get	- mHealth users become dependent on apps for
buggy only after developer updates	health needs and hence all functionalities need
	stringent testing across multiple devices before
	releasing updates

#### Compatibility

mHealth apps should be compatible with most devices to allow everyone to use them since they are improving and saving lives. A wide range of mHealth apps depend on external devices or trackers, such as smartwatches, and monitoring devices to track the activity of users. However, many mHealth app users have experienced compatibility issues when connecting external devices since they were not entirely supported, are old, or do conflict with other external devices. In addition, updates made to mHealth apps to introduce new features, fix existing issues, or enhance performance may compromise the apps’ capacity to work with external devices. According to app reviews, mHealth app users notice compatibility concerns after updating their apps to the latest version, which affects their usage of the app and even prevents some users from continuing progress in achieving their fitness goals. Some of these users were actually forced to update their phones’ firmware to use these apps, and then later complained that some developer upgrades damaged app compatibility with devices. While others updated their reviews to say their app compatibility issues were resolved after switching to the latest version. Moreover, many users noticed that various mHealth apps are incompatible with older mobile firmware, preventing some users from improving their health just because they own an old phone. Users with older phones that do not support the latest OS have also reported that some app updates have rendered certain apps inaccessible. Fitness activity tracking, Patient health tracking and self-monitoring, and Education and Information apps were the most highly affected subcategories by compatibility issues from our user app review analysis. Table 8 summarises key issues with compatibility from our automated and manual user review analysis, and some recommendations for improved compatibility in mHealth apps based on this user feedback.

**Table 8:** Key issues and recommendations for mHealth app compatibility issues

Key issues found	Recommendations
- Developers not implementing impor-	mHealth app users often use apps under very
tant UI requests by elderly/disabled	stressful conditions e.g. managing severe illness
users	–key UI/UX issues reported need to be priori-
	tised by developers
- Changing app interface frequently or	- Implementing simple designs for mHealth
implementing complex designs	apps
- One interface for all users	- mHealth users are very diverse – need to al-
	low users to switch between / configure inter-
	face depending on their diverse needs and ex-
	periences
- Issues with old firmware versions and	- Many mHealth users have old handsets – de-
updates	velopers need to test UI thoroughly on differ-
	ent devices with different operating systems,
	screen sizes, etc before releasing updates

#### Updates

mHealth apps should be well tested before being released to the public. When app developers update mHealth apps to bring new features or to correct some existing bugs, they can cause more problems than benefits, such as stress and frustration for some users. During our manual analysis of mHealth app reviews, we found that users who updated their apps were either highly satisfied or not. That is evidenced by the fact that 33.93% of users, who raised update issues in their reviews, rated the apps one star, and only 26.15% rated them five stars. This shows that quality assurance (QA) engineers do not entirely test all new or existing features and functionalities of mHealth apps before releasing updates to users. Moreover, users were requesting app developers to offer new features, address bugs, and improve accessibility. Surprisingly, several of these requests were repetitive, which indicates that app developers either did not see these reviews or ignored them. Fitness activity tracking, Education and Information, and Patient health tracking and self-monitoring apps were the most highly affected subcategories by update issues from our user app review analysis. Table 9 summarises key issues with update from our automated and manual user review analysis, and some recommendations for handling update issues in mHealth apps based on this user feedback.

**Table 9:** Key issues and recommendations for mHealth app update issues

Key issues found	Recommendations
- mHealth apps suffering from major	- Testing mHealth apps with realistic range of
bugs after being updated	end user devices, usage scenarios before releas-
	ing updates
- Users requesting updates to add/re-	- Proactive monitoring, prioritisation and han-
move mhealth features and function-	dling of update requests
alities, fix some bugs with analysing	
health data, or increase accessibility	
- Repetitive update requests for the	- Better triage of requests to address common
same issue from different users	update requests raised by different users

#### Connectivity

Most mHealth apps require internet access or communication with other devices to function as intended. mHealth app users’ may encounter some difficulties or problems while using the app if the app loses the internet connection. According to user reviews, several mHealth apps rely on the internet to work and are rendered useless if the network connection is lost. Some users stated that they did not get any messages about maintenance to become aware that they would not be able to use the app at that time. According to user review analysis, some mHealth apps do not completely support external devices like smartwatches, preventing users from using them and enjoying the full app experience. Moreover, some users stated that after updating their apps, their external devices became unsupported. Before downloading or purchasing mHealth apps, users must be informed of all compatible external devices. Also, app developers should test new software updates on multiple devices before releasing them to the public. Fitness activity tracking, Emerging, and Patient health tracking and self-monitoring apps were the most highly affected subcategories by connectivity issues from our user app review analysis. Table 10 summarises key issues with connectivity from our automated and manual user review analysis, and some recommendations for improved use of connectivity in mHealth apps based on this user feedback.

**Table 10:** Key issues and recommendations for mHealth app connectivity issues

Key issues found	Recommendations
- Some analysis and training mHealth	- Showing an internet connection indicator or
features are dependent on the internet	status while processing. Also showing an error
if not properly connect they will pro-	message and delete the data once the internet
vide wrong outcome and recommenda-	is down so user an start again on recovery
tion	
- Apps not supporting different types	- Stating the supported external medical de-
of external medical devices/trackers	vices/trackers in the app description
- Apps not connecting with specific	- Testing top used external medical devices/-
types of external medical devices/-	trackers properly before releasing app updates
trackers after users update their apps	
- Performing maintenance or updates	- Informing users about any scheduled main-
for mHealth apps that may affect	tenance or updates that may affect their con-
users’ connectivity without informing	nectivity within the app, that way they can
them earlier	reschudule work and appointments with clients

#### Internationalisation

Since users of mHealth apps are of different ages, genders, and cultures, these apps need to be developed and designed to be accessible by all types of users. By looking into mHealth user reviews, it was clear that mHealth app users face considerable challenges when they are unable to use the app in their language. User dissatisfaction grew when the app did not support their language. Reviewers say they feel more at ease using an app in their own language, and they requested the apps be supported in different languages. mHealth apps should offer the same functionalities and features to all users, regardless of their location or spoken language. In the user reviews, some users reported that the app’s interface and functionalities varied depending on the language used. Some users also noted translation issues that can massively affect the lives of people if they get the wrong information. On the other hand, some mHealth users expressed their satisfaction that the app supports their native language. Mental Health, Education and Information, and Lifestyle Planner and Goal Tracker apps were the most highly affected subcategories by Internationalisation from our user app review analysis. Table 11 summarises key issues with internationalisation from our automated and manual user review analysis, and some recommendations for handling internationalisation issues in mHealth apps based on this user feedback.

**Table 11:** Key issues and recommendations for mHealth app internationalisation features

Key issues found	Recommendations
- Some mHealth functionalities suffer	- As mHealth app users are diverse in terms of
from some translation issues	language, more thorough internationalisation
	including medical terms and health expecta-
	tions of divere users before releasing updates
- Users request updates to add lan-	- Developers should ensure that the devel-
guage support or medical/health ex-	oped app supports the widely used lan-
pressions from their own language to	guages, including using the most common
make the app more accessible	medical/health expressions in different lan-
	guages
- Some apps target a specific age or	- Developers should ensure that the app tar-
gender, which benenefits a particular	gets users of wide range of ages and genders,
type of user and is a concern for others	unless the app is targeting a specific health is-
	sue relevant to a particular group

#### Account and Logging

To allow mHealth app users to have a more personalised experience and better security, signup and login are required for many of these apps before users can start using them. Some reviewers stated the registration process was difficult, making some apps inaccessible. The signing up and logging in process should be easy and straightforward since many users of these apps are elderly and do not have a good technical background. Some mHealth users have reported in their reviews several issues affecting the logging process, including too many details or information required to sign up, errors in the registration form or login issues for various reasons. Emerging, TeleHealth and telemedicine, and Education and Information apps were the most highly affected subcategories by account and logging issues from our user app review analysis. Table 12 summarises key issues with account and logging from our automated and manual user review analysis, and some recommendations for improved use of account and logging in mHealth apps based on this user feedback.

**Table 12:** Key issues and recommendations for mHealth app logging issues

Key issues found	Recommendations
- Complex or buggy signup health	- Implementing simple sign up health forms
forms where users need to complete	and ensuring that the form is bug free. Along
and submit too many health details or	with some integration to get the latest and
information or face errors while enter-	most accurate users health details which comes
ing their health data within the regis-	from other areas such as hospitals and GP clin-
tration form	ics
- Verification codes or OTP are not	- Implementing different ways for users to ver-
properly delivered	ify their accounts besides OTP
- Incorrect verification codes where	- Ensuring sending unexpired verification
users get errors when submitting the	codes to users to complete the signup process
verification code they have received	
- Users not being able to retrieve their	- Allowing users to retrieve their user name in
users’ names	case they forgot it
- Users cannot reset their passwords	- Allowing users to sign in to the app using a
since forgot passwords functionality is	passcode or face id
not working properly	
- The app keeps signing out, requiring	- Allowing users to keep being signed in as long
users to log in each time	as they did not sign out
- Users are trying to log in while the	- Allowing users to access the app status in
app is temporarily down without hav-	case the app is down
ing any information about the app er-	
ror, so they end up resetting their ac-	
count information	

#### Notifications

Many mHealth apps utilise notifications to remind users of important updates or to prompt them to take immediate action regarding their health. Informative, timely, personalised, and relevant push notifications can help developers boost user satisfaction and improve users’ health. It is frustrating and distracting for mHealth users when apps deliver too many notifications, especially with premium subscription offers. According to reviews, some mHealth app developers were able to obtain enough data about their users’ interests to tailor their notifications to them. Users of fitness and health apps greatly appreciated personalised notifications, which allowed them to take immediate action to achieve their fitness goals. There were several complaints about some apps sending the same notification to several users. Our user review analysis indicated that developers must ensure that their app’s notifications are distinctive and depend on the user’s activity. mHealth app developers can offer users the option to opt-in for a weekly or monthly generic non-personalised notice to avoid irritating them with useless notifications. Scheduling and reminders, Emerging, and Lifestyle Planner and Goal Tracker apps were the most highly affected subcategories by notification issues from our user app review analysis. Table 13 summarises key issues with notification from our automated and manual user review analysis, and some recommendations for improved use of notification in mHealth apps based on this user feedback.

**Table 13:** Key issues and recommendations for mHealth app notification features

Key issues found	Recommendations
- Non-useful and non-personalised	- Testing with variety of end user groups to
health notifications	ensure useful, timely, personalised and relevant
	notifications to users
- Large number of notifications pushed	- Allow users to control notifications; design so
	that less pushed notifications to users
- The inability to choose health notifi-	- Allowing users to manually select the type
cation content by users	of health-related notifications they wish to re-
	ceive
- Notifications containing poor/inap-	- Testing processes focusing on detecting
propriate content	poor/inappropriate content and use of too
	generic notifications

#### Privacy

Many mHealth apps require users to grant some permissions in order to function properly, especially since most of them aim to track users’ activities, location, etc. Granting some of these permissions can compromise users’ privacy by accessing sensitive data such as their location, contacts, photos, and interactions with other apps. Worryingly, our manual user review analysis reveals that many mHealth app users were puzzled as to why their mHealth apps asked for some of these permissions when they were not required for the functionality of the app. Moreover, some users provide location tracking permission without understanding what happens behind the scenes since they cannot read the whole privacy policies for these apps. Emerging, Education and Information, and Fitness activity tracking apps were the most highly affected subcategories by privacy issues from our user app review analysis. Table 14 summarises key issues with privacy from our automated and manual user review analysis, and some recommendations for handling privacy in mHealth apps based on this user feedback.

**Table 14:** Key issues and recommendations for mHealth app privacy issues

Key issues found	Recommendations
- Apps gaining access to non-essential	- Only request access to essential health data
health data or features such as users’	or features that massively affect and enhance
contacts, cameras, photo galleries, mi-	the app’s usage and allowing users to grant
crophones, location, local network de-	or reject permission and to have the option to
vices, Bluetooth, and interaction with	change their minds later on
other apps	
- Apps asking for users’ private infor-	- Do not ask users to provide personal infor-
mation, such as their date of birth	mation unless it affects the app’s functionality
- Apps forcing users to accept their	- Simplifying privacy policies and data user
lengthy and complicated privacy poli-	agreements for users
cies before they can use them	
- Apps frequently accessing users’ lo-	- Accessing user’s location only if needed
cations in the background	

#### Uninstallation

To improve app acceptance, mHealth app developers typically intend to develop apps that improve or save the lives of their users. This is done by developing updates to allow both stability and feature-richness. mHealth app users may delete or uninstall these apps if they fail to achieve this purpose. Having severe problems or instability issues is reported in user reviews as a significant reason for deletion. Users stated in their reviews that several of these issues/problems only appear after app updates, which affect their current progress and prevent them from achieving the goal of downloading these apps. Moreover, using an mHealth app with severe accessibility problems can prompt users to delete it as some mHealth users reported serious accessibility problems within the app, forcing them to delete it. Before downloading an mHealth app, users should be transparently informed about its functionality and features. According to user reviews, when an mHealth app does not meet their expectations, users delete or uninstall it. One of the common reasons that users uninstall mHealth apps is that significant functionalities and features require in-app purchases. This arises when app developers do not properly describe the free edition’s features in the app description. Some users also note that the mHealth app screenshots in the description do not resemble reality. Emerging, Fitness activity tracking, and TeleHealth and telemedicine apps were the most highly affected subcategories by uninstallation issues from our user app review analysis. Table 15 summarises key issues with uninstallation from our automated and manual user review analysis, and some recommendations for handling uninstallation issues in mHealth apps based on this user feedback.

**Table 15:** Key issues and recommendations for mHealth app uninstallation issues

Key issues found	Recommendations
- Inaccessible mHealth apps	- Including diverse user personas, focus groups
	and testing with diverse end users to ensure
	better accessibility within apps
- Poorly described mHealth apps	- Testing app descriptions with diverse end
	users as well as app itself
- mHealth Apps having major stability	- as mHealth apps may be critical to quality
issues or bugs	of life / health, rigorous testing processes be-
	fore release is arguably more important than
	in many other app domains

#### Advertising

Advertisements (ads) are needed by many mHealth apps to monetise them and create an extra stream of revenue for developers, especially for free ones. mHealth app users can become unsatisfied with the way ads are implemented in the app and the ad content, which negatively affects their overall experience with continued usage of the app. Some mHealth app users indicated in their reviews that they have issues with such advertisement-driven revenue making. These include issues with annoying ads, too many ads, ads not related to the purpose of the app, too big ads, and inappropriate or offensive ads, which distract mHealth app users from the goal of downloading and installing these apps and lead them to miss important notifications to improve their health. Patient health tracking and self-monitoring, Fitness and workouts, and Lifestyle Planner and Goal Tracker apps were the most highly affected subcategories by advertising issues from our user app review analysis. Table 16 summarises key issues with advertisements from our automated and manual user review analysis, and some recommendations for improved use of ads in mHealth apps based on this user feedback.

**Table 16:** Key issues and recommendations for mHealth app advertising features

Key issues found	Recommendations
- Inappropriate ad content unrelated to	- Ensuring that all content of the ads shown in
Health issues	their app is appropriate for the target users of
	the app; Allowing op-out of some ad domains
- Ads that do not match mHealth app	- Ensuring that the content of the ads appear-
goals/purpose	ing in their mHealth app matches the goal of
	the app
- Frequent and lengthy Health im-	- Decreasing ad frequency and length as much
provement medication ads	as possible
Ads affecting functionality of the	- Showing ads in the right position and min-
mHealth app	imising ad size
- Health and medical ads that are non-	- Ensuring that ads do not affect functionality
skippable	of the app
- Health and medical ads position and	- Allowing users to skip ads
style	
- Health and medical ads covering large	- Minimising size of ads
space of the screen	
- Health and medical ads volume and	- Allowing user control of ad volume
distraction	

#### Resources

Some mHealth apps are designed to be used for long periods of time by their users for activity tracking, workout guidance, etc. This results in battery drainage becoming a typical and unpleasant resource issue when being used. According to user reviews, battery drainage issues are widespread in mHealth apps that need prolonged use, which leads users to reduce app usage, which affects their goal of installing the app. Some users of fitness tracking apps say they need to connect a battery bank to keep their phones charged during exercise since these apps use a lot of computing power due to the continuous usage of the global positioning system (GPS). Others link this issue to mHealth apps that track or check users’ locations or apps that run in the background and deliver frequent notifications. Due to battery drainage problems, some mHealth app users have discontinued or restricted some or all of the permissions of their apps. Another reported issue was the large amount of space or memory mHealth apps require to be installed and run correctly. Based on user reviews, we found that users with old phones or limited storage were more prone to resource concerns when using mHealth apps. Moreover, some apps take up a lot of phone storage, and this problem persists and gets worse following app updates. Others reported being unable to update apps due to a lack of space on their phones. Users of mHealth apps on old phones with poor computational power have major concerns about slow processing, lagging, and freezing. Emerging, Sleep and meditation, and Fitness activity tracking apps were the most highly affected subcategories by resources issues from our user app review analysis. Table 17 summarises key issues with resources from our automated and manual user review analysis, and some recommendations for improved use of resources in mHealth apps based on this user feedback.

**Table 17:** Key issues and recommendations for mHealth app resources issues

Key issues found	Recommendations
- mHealth apps do processing and	- Allow the mHealth app to work in the back-
analysis in the background cause slowing down of phones and increasing battery drainage	ground only if needed
- mHealth apps that regularly perform	- Ensuring that the mHealth app only performs
functionalities and high computations	tasks that may cause battery drainage only if
that lead to fast battery drainage, such	required
as accessing user location, pushing lots	
of notifications, etc	
- mHealth apps that need large free	- Release apps’ updates with a small size so
space to be downloaded or updated	that all users owing phones with different free
	space can download them
- mHealth apps consuming massive	- Ensure that the app does not consume high
memory while being used	computational resources before releasing the
	mHealth app to users

### Implications for Researchers

#### Generalisation

Our data analysis allows researchers to identify the major issues raised in user reviews of mHealth apps based on over 5.1 million reviews with different 14 aspects and work to address them. Our tool and techniques can be applied to other mHealth apps, but also to other categories of apps to see if they differ in frequency of issues or face similar issues to mHealth apps.

#### User Location Impact

Our analysis will also allow researchers to explore how users’ ratings might differ from one language to another and/or from one country to another. Similarly, we can compare Android and iPhone app versions to see if there are any marked differences in user feedback and explore why.

#### App Evolution

Researchers can also use our approach to investigate the version histories of mHealth apps and seeing if the developers of mHealth apps were able to fix the issues that were repetitively raised by users in their regular updates.

#### Design guidance

As discussed in the previous section, our analysis has identified a range of common issues with mHealth apps. A set of guidelines for mHealth app developers to follow while building or updating mHealth apps could be developed. This would help and facilitate mHealth app development organisations and companies to proactively discover and prevent software and design issues before their final apps are released to mobile users based on some recommendations.

#### Targeted Evaluation

Early discovery of mHealth app issues by developing a proactive evaluation model could save a large amount of money. It could also make mHealth apps more effective and engaging, and thus even contribute to reducing the 3.2 million deaths every year due to inactivity. Moreover, an increase in reliability and credibility will lead to a higher download rate of these apps when users feel that the updates are really fixing their issues.

### Threats to Validity

#### Internal

Our technique is dependent on automated review analysis, where a large word dataset helped us categorise user reviews into 14 aspects. We correlated this classification with the overall app rating submitted by the users. Some of our words/phrases may be wrong or lack some essential words mentioned in user reviews. To eliminate this issue while creating the classification dataset, we manually reviewed over 23,000 reviews from a wide range of apps, not just mHealth apps. Moreover, since we rely on Google’s API for translating non-English user reviews to English, minor translation errors can lead to incorrect categorisation and, in some cases, un-classifying some reviews which should have been classified into a specific aspect. This also applies to reviews that have spelling mistakes, where our tool can not either correctly translate or classify. In addition, some of the words used in our classification might be used in different contexts where the review is classified incorrectly into a specific category. We tried to eliminate these issues by using clear words or phrases which have low ambiguity. Furthermore, we manually reviewed and analysed some of the reviews after the classification to highlight and report some of the commonly raised issues. This does not guarantee that we have noted all the issues and problems raised by the users in their reviews since we were analysing over 5 million reviews. In addition, we only included mHealth apps having more than 500 reviews.

#### External

Despite analysing over 5.1 million reviews for 278 mobile apps, our analysis is still limited to mHealth apps. The findings and results of our study do not reflect all categories of mobile apps. We could not generalise our results to other categories of mobile apps. We predict different outcomes from those we are presenting now by conducting the same analysis on a different category of mobile apps, such as social media apps. We only got data from one source, which is user reviews. We have considered the psychological realism of all the reviews given. We are lucky in this case as the data was already provided by the user as feedback to the app developers and not as a part of a certain survey that might be biased. That helped us as this data reflects the real world problem, not a cover story.

#### Construct

In some reviews, users report multiple issues and problems within the same review. Users can only submit one rating in their review, despite the fact that they might be expressing both good and negative issues in the same review. Having more than one aspect in the same review will lead these reviews to be classified into more than one aspect while having the same rating in all classified aspects. This could lead to slight adjustments in our overall ratings. In practice, during manual review we found very few reviews that contained mixed positive and negative feedback. In addition, we have not yet shared the results of our analysis with developers to ask them whether it helps them while developing or updating mHealth apps.

## Related Work

mHealth apps are downloaded and used by millions of people around the world on a daily basis. There are currently more than 318,000 fitness and health apps in the mobile app market (Liew et al. 2019; Institute 2020). When interacting with mobile apps, users from diverse cultural backgrounds have different preferences (Oliveira et al. 2016; Reinecke and Bernstein 2011; Reinecke and Gajos 2014). That is why the feedback from users across different cultures and languages differs for the same app (Guzman et al. 2018). As shown in Anderson et al. (2016), many mHealth apps are buggy, in which their users lose engagement and discontinue using them after a short period of time. Many users are experiencing some issues while using the mHealth apps and want to submit some feedback to the developers of these apps. There are many ways for users to submit their feedback to mobile app developers for development and research purposes such as contact forms and mobile surveys, emails and social media feedback, and user reviews. On the other hand, mobile app development organisations are always seeking users’ feedback. This feedback allows the developers of these organisations to enhance their apps with their regular updates. Surprisingly, 42% of organisations and companies fail to analyse users’ feedback due to some challenges. Not listening to users’ feedback and experiences leads to missing an excellent opportunity to improve apps and enhance users’ satisfaction and experience.

A number of studies have investigated and analysed user reviews for mobile apps. Several studies looked into different methodologies to analyse user reviews for extracting app features (Guzman and Maalej 2014; Iacob and Harrison 2013). Other studies developed some methods to classify user reviews into predefined aspects (Haggag et al. 2022; Fazzini et al. 2022; Chen et al. 2014; Haggag et al. 2021; Gomez et al. 2015; Gu and Kim S 2015; Maalej and Nabil 2015; Haggag 2022). Moreover, some research has been conducted on mHealth apps, but they concentrate only on user experience, neglecting the users’ dedication to the apps. In Di Sorbo et al. (2021), the authors developed a tool called NEON that use a natural language processing approach. They did a small study on 100 reviews across two categories (feature request and problem discovery). The authors picked up around 35% of the rules inferred by their tool are relevant for identifying app review sentences, reporting bugs or requesting enhancements. The authors indicated that the effectiveness of NEON would degrade when dealing with review sentences containing mixtures of code elements and natural language or incomplete sentences. In our study, we had many user review sentences that were not complete, had spelling errors, were written in different languages, etc. In Pimenta et al. (2020), the authors tried to design mechanisms to improve and introduce a healthier behavioural change through mobile devices. They detected the experience satisfaction using BCT Taxonomy and user feedback. They had done a systematic review of the applications, which was followed by sentiment analysis. Then finally, they came up with the design implications, which are related to the implementation of BCT features. That interactive system has shown various relationships, such as being physically active and having the habit of eating healthy, while negative behaviours were mostly triggered by rewards and threats. In Alqahtani and Orji (2020), the authors were focusing on Mental health applications, as it reflected a great intervention on mental issues. Their findings showed that the mobile interventions’ effectiveness led to low engagement, and they failed to understand the reasons for this behaviour. However, the authors discovered two very important factors, user usability and experience. They were trying to analyse user reviews to understand the reasons behind users’ ceasing the use of these applications. They have reviewed 106 mental health, 13,549 reviews. They pointed out that the main reasons for quitting the usage of apps are the user interface and user-friendliness. A long-term commitment to using the app does not necessarily mean involvement (Zichermann 2011; Pandey et al. 2013). Since the mobile apps market is highly competitive, apps’ developers need to take their users’ feedback seriously, not to lose any of their loyal users. This can be done by carefully analysing users’ reviews and working on resolving their issues and fulfilling their needs (Khalid et al. 2014). Ratings are always associated with the quality and popularity of the mobile app (Khalid et al. 2014). Mobile users generally see a mobile app with a low rating as non-popular, which badly affect the number of downloads and revenues (Khalid et al. 2014). The number of user reviews increases when the number of downloads increases (Mcilroy et al. 2017). Moreover, the number of user reviews is directly correlated with the number of apps updates done by the developers of mobile apps (Mcilroy et al. 2017). This leads to a spike in the number of reviews after each update as users are either experiencing new problems while using the app, or they are fully satisfied with the new update (Mcilroy et al. 2017). In Vasa et al. (2012), an exploratory study was performed on what could impact the users to write an app review. In Iacob et al. (2013), they investigated different factors that affect user reviews, such as the correlation between the rating, price, and the number of downloads. The authors of (Chen et al. 2014), proposed a computational structure by adopting a semi-supervised algorithm in order to extract and rank insightful reviews. In Vu et al. (2015), the authors developed a semi-automated keyword-based approach for mining user opinions in their reviews. In Mcilroy et al. (2017), the authors indicated that further studies are needed to better understand and analyse user reviews through Apple and Google app stores. More research has been done on data reviews, in Wu et al. (2021) they tried to identify the key features from app user reviews. In Di Sorbo et al. (2021), they have built an NLP-based tool for software artefact analysis. In Di Sorbo et al. (2016) and Panichella et al. (2015), the authors came up with a list of recommendations for software changes, including maintenance and evolution. Also, some research has been done on profiling users via their reviews and doing that via a systematic approach as shown in Dong et al. (2021) and Genc-Nayebi and Abran (2017).

## Conclusions

The global market for mHealth apps is increasing at a rapid pace, resulting in significant revenues and downloads. However, as reported in user reviews, many of these mHealth apps have significant problems. App developers would benefit from a better understanding of major user concerns in order to improve the quality and adoption of their apps. While many previous studies have employed app reviews to investigate users’ opinions about mHealth apps, many of them are limited in scope, scale, and/or analysis. In this paper, we analysed over 5 million user reviews of 278 mHealth apps. We classified these reviews into 14 different aspects/categories of issues reported using an automated tool that we developed based on a bag of keywords. User satisfaction levels were compared amongst several mHealth app subcategories to investigate the impact of different aspects of mHealth apps on their ratings. Based on our findings, women’s health apps were recognised as the highest-rated subcategory. In contrast, fitness activity tracking was the lowest-rated app subcategory in our review of all mHealth subcategories due to various issues and problems in multiple investigated aspects. Over half of the users who reported uninstallation issues in their reviews rated the app 1-star. Half of the users rated the account and logging aspect 1-star each due to several problems and issues they faced while signing up or logging in to their account. Over a third of users who raised privacy concerns rated the app a 1-star review. However, only 6% of users gave the app a 1-star rating for UI/UX. 20% of users reported issues with the handling of user requests and internationalisation concerns. By manually analysing a sample of 1,000 user reviews from each investigated aspect/category, we validated our findings. We developed a list of recommendations for mHealth app developers for each investigated aspect based on our user review analysis.

## Footnotes

1 Access by: https://www.statista.com/
